# Birds from the Azores: An updated list with some comments on species distribution

**DOI:** 10.3897/BDJ.3.e6604

**Published:** 2015-11-20

**Authors:** Luís MD Barcelos, Pedro R Rodrigues, Joël Bried, Enésima P Mendonça, Rosalina Gabriel, Paulo Alexandre Vieira Borges

**Affiliations:** ‡ CE3C – Centre for Ecology, Evolution and Environmental Changes / Azorean Biodiversity Group and Universidade dos Açores, Azores, Portugal; § Universidade dos Açores, Ponta Delgada, Portugal; | na, Biarritz, France

**Keywords:** Azores, birds checklist, species distribution

## Abstract

**Background:**

An updated checklist of the Birds of the Azores is presented based on information compiled from Rodrigues et al. (2010) and from the websites, Azores Bird Club. (2014), Aves dos Açores (2014) Azores Bird Sightings (2014) and Vittery (2014), since 2010.

**New information:**

The checklist has a total of 414 species, including 38 new species.

Almost half of the species and subspecies that occur in the Azores have a Palearctic origin, the remaining ones being essentialy Nearctic and Holarctic species. São Miguel is the island with the highest number of bird species, followed by Terceira, Corvo and Flores islands.

## Introduction

Birds (Vertebrata: Aves) are some of the most iconic animals. They play important roles in the ecosystem, and since they are abundant and diverse in most urban and rural areas, humans have established a good long-lasting relationship with them (e.g., birdwatching, photography). However, amongst the ca 10.000 bird species which have been living on Earth since the appearance of modern humans, many species were regionally lost or extinct, or are endangered, especially on islands (Sax et al. 2002, Elphick et al. 2010, Rando et al. 2013, Alcover et al. 2015). Updating a list of birds from remote oceanic islands represents an important step towards an improved knowledge of colonization mechanisms and migratory patterns by animals and a contribution towards the conservation and management of insular taxa and their natural habitats.

**Figure 1.**
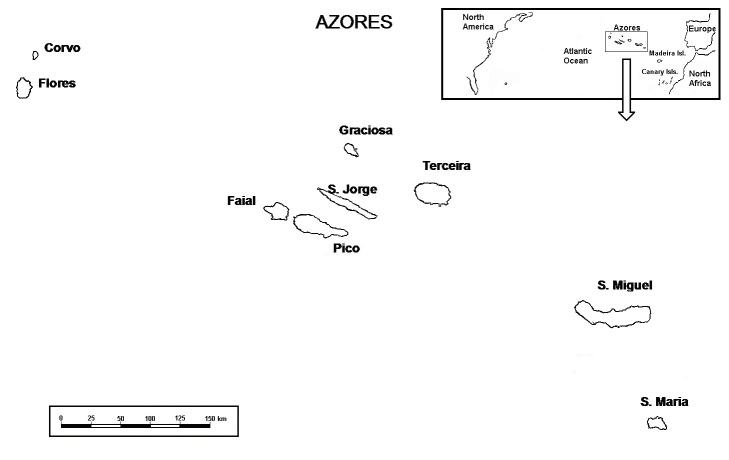
Map of Azores islands, Portugal (Modified from Amorim 2005).

The Azores Archipelago is located in the North Atlantic Ocean, between 36°55’ and 39°43’ N, and 24°46’ and 31°16’ W, about 1500 km from mainland Europe and 1900 km from North America (Fig. 1). The Azores is a volcanic oceanic archipelago consisting of nine islands and several islets of recent volcanic origin (0.25 to 8.12 My old), which are spread over >600 km along a northwest-southeast transect (França et al. 2003). The oceanic distribution of these islands and, as a consequence, their relative positions, has led to the formation of the following groups: (i), a western group, comprising the islands of Flores and Corvo; (ii), a central group, with the islands of Faial, Pico, São Jorge, Terceira and Graciosa and (iii), an eastern group, made up of the islands of São Miguel and Santa Maria.

Due to its remoteness, the Azores archipelago was discovered and humans settled only at the beginning of the fifteenth century. The settlers described the islands as covered by dense native vegetation, with countless birds, especially seabirds (Frutuoso 2005a, Frutuoso 2005b, Frutuoso 2005c). Until then the archipelago was definitely a bird paradise, not only for seabirds that could use the vast coastal cliffs of the islands and islets to breed, but also for terrestrial birds which were widespread in the lush Azorean forests (Rodrigues and Cunha 2012). After human colonization started in 1439, several bird species suffered a sudden decrease in population size due to their use as food and oil source for human consumption, as well as to the introduction of predators and the destruction of their habitat (Rodrigues et al. 2010). As a consequence of the human impact, six flightless bird species are confirmed to be extinct in the Azores, namely one *Otus* species (Rando et al. 2013) and five *Rallus* species (Alcover et al. 2015), but recent findings indicate that many more will be described soon (Josep Alcover and Juan Rando, pers. comm.).

**Figure 2.**
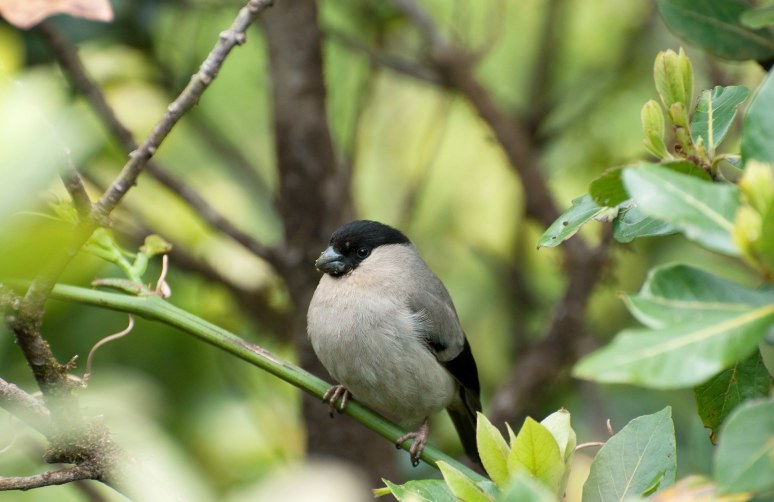
Azores Bullfinch (*Pyrrhula murina* Godman, 1866). Photo by PAV Borges (2008).

**Figure 3.**
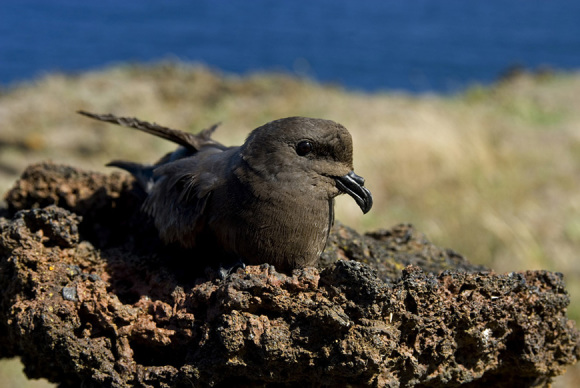
Monteiro’s Storm Petrel (*Oceanodroma monteiroi* Bolton et al., 2008). Photo by PH Silva (2014).

Despite the reduction of several bird populations, the Azores remain of great interest for ornithologists and are considered as one of the most popular destinations for European birdwatchers (Pereira 2010, Rodrigues and Michielsen 2010), all the more since they hold two endemic species: the Azores Bullfinch (*Pyrrhula murina* Godman, 1866) (Fig. 2), and Monteiro’s Storm Petrel (*Oceanodroma monteiroi* Bolton et al. 2008) (Fig. 3), and 11 endemic subspecies. Besides, the Azores archipelago displays colonies of several seabird species of great importance in the European context, and it is also possible to observe pelagic birds and a large variety of migratory birds, some of which are very rare Nearctic vagrants to the western Palearctic (Rodrigues and Michielsen 2010, Barreiros et al. 2014).

Rodrigues and Cunha (2011) resumed the ornithological history of the Azores, from the beginning of human settlement until recently, and the latest checklist of birds from the Azores archipelago was published in 2010 by Rodrigues et al., as part of the complete list of Azorean biota published by Borges et al. (2010). This list was based on an exhaustive review of the published literature (e.g., Le Grand 1993, Monteiro et al. 1996, Monteiro et al. 1999, Elias et al. 2003, Elias et al. 2004, Elias et al. 2005, Elias et al. 2006, Bried et al. 2007, Jara et al. 2007, Jara et al. 2008, Bolton et al. 2008, Imber 2008, Vittery 2014) but also on unpublished data following Rodebrand (2010) and Rodebrand S. & The Birding Azores Team (2010). This checklist totalled 384 species, distributed between three categories: breeding, non-breeding and potentially breeding (species reported as introduced and/or escapes from captivity and for which suitable nesting conditions exist in the Azores, but whose numbers are insufficient to allow the maintenance of self-sustaining populations in this archipelago). During the last years, and due to the growing interest of birdwatchers in the Azores avifauna, several dedicated websites have been developed, such as ABC – Azores Bird Club. (2014) (formerly Birding Azores) (http://www.birdingazores.com/), AdA – Aves dos Açores (2014) (http://azores.avesdeportugal.info/) and ABS – Azores Bird Sightings (2014) (http://azoresbs.weebly.com/ from January 2012 to February 2014 and http://azoresbirdsightings.blogspot.co.uk/ from August 2014 onward). These websites are presently used by birdwatchers as a tool to register new information about species occurrence and distribution in the Azores.

The present paper updates the previous checklist (Rodrigues et al. 2010), with respect to species distribution within the archipelago and also by including new species to the archipelago. The updating is based on the sightings reports available on the websites mentioned above. We also perform a nomenclatural review of the Orders, Families, Species and Subspecies, following the 2014 IOC World Bird list (v 4.3) (Gill and Donsker 2015) published by the International Ornithologists’ Union (available at http://www.worldbirdnames.org/). Some additional notes on the distribution of the different species among the islands and their biogeographic origin are presented.

## Materials and methods

Based on Rodrigues et al. (2010), we completed a full taxonomic revision using the IOC Bird list (Gill and Donsker 2015) as a reference to identify the changes which occurred in the taxonomic hierarchy and synonymies. Then, we updated the list of the species recorded in the Azores archipelago until the end of 2014, as well as their distribution within the archipelago. The species newly reported for the Azores are signalled as NSR (New Species Record). Most of these NSRs were reported after 2010, but there are also a few missing records prior to this date. The species previously reported by Rodrigues et al. (2010), but whose distribution has changed since then, are signalled with an asterisk (*). All the new information about species occurrence and distribution was compiled based on the birdwatching websites mentioned earlier, and relies on a large number of birdwatcher contributions.

In order to make the data easier to consult, the main checklist (Checklist 1) includes all the breeding and non-breeding species together, including the escapes and/or introduced species which already have feral populations in the Azores. All the other escapes and/or introduced species were excluded from the main list, and can be consulted in Checklist 2.

We considered the species with five our less records, in Azores, as Rare Species. This species are presented in a table (Suppl. material 3) with the number of sightings, number of individual recorded, and some notes.

**Figure 4.**
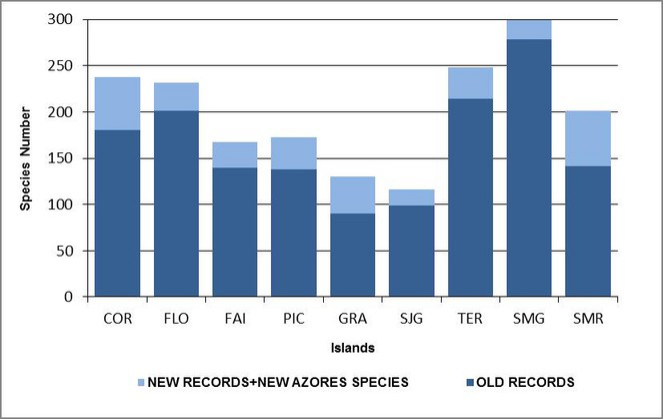
Global species richness on the nine Azorean islands (COR= Corvo; FLO = Flores; FAI = Faial; PIC = Pico; GRA =Graciosa; SJG = São Jorge; TER = Terceira; SMG = São Miguel; SMR = Santa Maria) (Suppl. material 1).

Besides the taxonomical information and the distribution among the Azorean islands (Fig. 4), we include information on: i) biogeographical origin, based on (Lomolino et al. 2010); ii) breeding status; iii) islands where breeding has been documented and iv) current protection status of the species according to the Azorean Decree DLR n.° 15/2012/A Governo dos Açores (2012) from 2nd April 2012, which transposes the European Directives (Birds), Conventions (Bern and OSPAR), Agreements (AEWA) and specific regional protection statuses (TOP100) to the regional legislation. The protection statuses are represented by a letter or code (corresponding to the directive, convention or law) and by a Roman numeral (corresponding to the annex) as follows: P – Priority species for conservation; A – Birds directive; B – Bern Convention; AEWA – African-Eurasian Waterbird Agreement; O – OSPAR Convention; T100 – 100 prioritary threatened species for management purposes in the European biogeographical region of Macaronesia (Martín et al. 2008); (X) – New species disaggregated from Madeiran Storm-petrel *Oceanodroma castro* (Harcourt, 1851) and which is known to breed only on Praia and Baixo islets (Graciosa Island).

## Checklists

### List of the non-breeding and breeding species from Azores

#### Chordata

#### Vertebrata

#### Aves

#### Anseriformes

#### Anatidae

#### Aix sponsa

(Linnaeus, 1758)

##### Ecological interactions

###### Native status

Nearctic

##### Distribution

COR; FLO; GRA; TER*; SMG

##### Notes

Occasional Migrant. Rodrigues et al. (2010)

#### Anas acuta

Linnaeus, 1758

##### Ecological interactions

###### Native status

Holarctic

###### Conservation status

A-IIA; AEWA

##### Distribution

COR; FLO; FAI; PIC; SJG; TER; SMG; SMR*

##### Notes

Regular Wintering. Rodrigues et al. (2010)

#### Anas americana

Gmelin, 1789

##### Ecological interactions

###### Native status

Nearctic

##### Distribution

COR; FLO; FAI; PIC; SJG; TER; SMG; SMR*

##### Notes

Regular Wintering. Rodrigues et al. (2010)

#### Anas carolinensis

Gmelin, 1789

##### Ecological interactions

###### Native status

Nearctic

##### Distribution

COR; FLO; FAI; PIC; TER; SMG; SMR*

##### Notes

Regular Wintering. Rodrigues et al. (2010)

#### Anas clypeata

Linnaeus, 1758

##### Ecological interactions

###### Native status

Holarctic

###### Conservation status

A-IIA; AEWA

##### Distribution

COR*; FLO; FAI; TER; SMG; SMR*

##### Notes

Regular Wintering. Rodrigues et al. (2010)

#### Anas crecca

Linnaeus, 1758

##### Ecological interactions

###### Native status

Palearctic

###### Conservation status

A-IIA; AEWA

##### Distribution

COR; FLO; FAI; PIC; GRA*; SJG; TER; SMG; SMR

##### Notes

Regular Wintering. Rodrigues et al. (2010)

#### Anas discors

Linnaeus, 1766

##### Ecological interactions

###### Native status

Nearctic

##### Distribution

COR; FLO; FAI; PIC; GRA; SJG; TER; SMG; SMR

##### Notes

Regular Wintering. Rodrigues et al. (2010)

#### Anas penelope

Linnaeus, 1758

##### Ecological interactions

###### Native status

Palearctic

###### Conservation status

A-IIA; AEWA

##### Distribution

COR; FLO; FAI*; PIC; SJG*; TER; SMG; SMR

##### Notes

Regular Wintering. Rodrigues et al. (2010)

#### Anas platyrhynchos platyrhynchos

Linnaeus, 1758

##### Ecological interactions

###### Native status

Holarctic

###### Conservation status

A-IIA; AEWA

##### Distribution

COR (Occasional Breeder); FLO (Occasional Breeder); FAI; PIC; GRA*; SJG; TER; SMG (Occasional Breeder); SMR

##### Notes

Regular Wintering. Rodrigues et al. (2010)

#### Anas querquedula

Linnaeus, 1758

##### Ecological interactions

###### Native status

Palearctic

###### Conservation status

A-IIA; AEWA

##### Distribution

COR; FLO; FAI; PIC; TER; SMG; SMR*

##### Notes

Occasional Migrant. Rodrigues et al. (2010)

#### Anas rubripes

Brewster, 1902

##### Ecological interactions

###### Native status

Nearctic

##### Distribution

COR; FLO (Occasional Breeder); TER; SMG

##### Notes

Regular Wintering. Rodrigues et al. (2010)

#### Anas strepera

Linnaeus, 1758

##### Ecological interactions

###### Native status

Holarctic

###### Conservation status

A-IIA; AEWA

##### Distribution

COR; FLO; FAI; TER; SMG

##### Notes

Occasional Wintering. Rodrigues et al. (2010)

#### Anser albifrons

(Scopoli, 1769)

##### Ecological interactions

###### Native status

Holarctic

##### Distribution

PIC*; TER; SMG; SMR

##### Notes

Occasional Wintering. Rodrigues et al. (2010)

#### Anser anser

(Linnaeus, 1758)

##### Ecological interactions

###### Native status

Palearctic

##### Distribution

COR; TER*; SMG; SMR

##### Notes

Occasional Wintering. Rodrigues et al. (2010)

#### Anser brachyrhynchus

Baillon, 1834

##### Ecological interactions

###### Native status

Palearctic

##### Distribution

FLO; PIC*; TER; SMG; SMR

##### Notes

Occasional Wintering. Rodrigues et al. (2010)

#### Anser fabalis

(Latham, 1787)

##### Ecological interactions

###### Native status

Palearctic

##### Distribution

COR; TER

##### Notes

Occasional Wintering. Rodrigues et al. (2010)

#### Aythya affinis

(Eyton, 1838)

##### Ecological interactions

###### Native status

Nearctic

##### Distribution

FLO; FAI; PIC*; TER; SMG; SMR*

##### Notes

Occasional Wintering. Rodrigues et al. (2010)

#### Aythya collaris

(Donovan, 1809)

##### Ecological interactions

###### Native status

Nearctic

###### Conservation status

A

##### Distribution

COR; FLO; FAI; PIC; SJG; TER; SMG; SMR

##### Notes

Regular Wintering. Rodrigues et al. (2010)

#### Aythya ferina

(Linnaeus, 1758)

##### Ecological interactions

###### Native status

Palearctic

##### Distribution

COR; FLO; TER; SMG

##### Notes

Occasional Wintering. Rodrigues et al. (2010)

#### Aythya fuligula

(Linnaeus, 1758)

##### Ecological interactions

###### Native status

Palearctic

##### Distribution

COR; FLO; FAI; PIC; SJG*; TER; SMG; SMR

##### Notes

Regular Wintering. Rodrigues et al. (2010)

#### Aythya marila

(Linnaeus, 1761)

##### Ecological interactions

###### Native status

Holarctic

##### Distribution

COR; FLO; FAI*; PIC*; SJG*; TER; SMG; SMR

##### Notes

Occasional Wintering. Rodrigues et al. (2010)

#### Aythya nyroca

(Güldenstädt, 1770)

##### Ecological interactions

###### Native status

Palearctic

##### Distribution

SMG

##### Notes

Occasional Wintering. Rodrigues et al. (2010)

#### Branta bernicla hrota

(Müller, 1776)

##### Ecological interactions

###### Native status

Holarctic

##### Distribution

COR; FLO; TER; SMG

##### Notes

Occasional Wintering. Rodrigues et al. (2010)

#### Branta canadensis

(Linnaeus, 1758)

##### Ecological interactions

###### Native status

Nearctic

##### Distribution

COR*; FAI*; PIC*; GRA*; SJG; TER; SMG; SMR*

##### Notes

Occasional Wintering. Rodrigues et al. (2010)

#### Branta leucopsis

(Bechstein, 1803)

##### Ecological interactions

###### Native status

Palearctic

##### Distribution

COR*; FLO; SJG; SMG

##### Notes

Occasional Wintering. Rodrigues et al. (2010)

#### Bucephala albeola

(Linnaeus, 1758)

##### Ecological interactions

###### Native status

Nearctic

##### Distribution

PIC; GRA; TER

##### Notes

Occasional Migrant. Rodrigues et al. (2010)

#### Bucephala clangula

(Linnaeus, 1758)

##### Ecological interactions

###### Native status

Holarctic

##### Distribution

COR*; SJG; TER; SMG

##### Notes

Occasional Migrant. Rodrigues et al. (2010)

#### Chen caerulescens

(Linnaeus, 1758)

##### Ecological interactions

###### Native status

Nearctic

##### Distribution

TER; SMG

##### Notes

Occasional Wintering. Rodrigues et al. (2010)

#### Clangula hyemalis

(Linnaeus, 1758)

##### Ecological interactions

###### Native status

Holarctic

##### Distribution

FAI*; PIC*; SJG*; TER; SMG

##### Notes

Occasional Migrant. Rodrigues et al. (2010)

#### Cygnus olor

(Gmelin, 1789)

##### Ecological interactions

###### Native status

Palearctic

##### Distribution

FAI; PIC; GRA; TER; SMG

##### Notes

Occasional Migrant. Rodrigues et al. (2010)

#### Dendrocygna bicolor

(Vieillot, 1816)

##### Ecological interactions

###### Native status

Afro-tropical

##### Distribution

SMG

##### Notes

Occasional Migrant. Rodrigues et al. (2010)

#### Lophodytes cucullatus

(Linnaeus, 1758)

##### Ecological interactions

###### Native status

Nearctic

##### Distribution

COR; FLO; SMG

##### Notes

Occasional Wintering. Rodrigues et al. (2010)

#### Melanitta nigra

(Linnaeus, 1758)

##### Ecological interactions

###### Native status

Holarctic

##### Distribution

FLO; FAI; TER; SMG

##### Notes

Occasional Migrant. Rodrigues et al. (2010)

#### Melanitta perspicillata

(Linnaeus, 1758)

##### Ecological interactions

###### Native status

Nearctic

##### Distribution

FLO; FAI; SJG; TER; SMG; SMR*

##### Notes

Occasional Migrant. Rodrigues et al. (2010)

#### Mergus merganser

Linnaeus, 1758

##### Ecological interactions

###### Native status

Holarctic

##### Distribution

GRA; TER*

##### Notes

Occasional Wintering. Rodrigues et al. (2010)

#### Mergus serrator

Linnaeus, 1758

##### Ecological interactions

###### Native status

Holarctic

##### Distribution

COR; FLO; FAI; PIC; TER; SMG; SMR

##### Notes

Occasional Wintering. Rodrigues et al. (2010)

#### Oxyura jamaicensis

(Gmelin, 1789)

##### Ecological interactions

###### Native status

Nearctic

##### Distribution

FLO; TER

##### Notes

Occasional Migrant. Rodrigues et al. (2010)

#### Somateria mollissima

(Linnaeus, 1758)

##### Ecological interactions

###### Native status

Holarctic

##### Distribution

COR; SMG

##### Notes

Occasional Wintering. Rodrigues et al. (2010)

#### Somateria spectabilis

(Linnaeus, 1758)

##### Ecological interactions

###### Native status

Holarctic

##### Distribution

SMG

##### Notes

Occasional Wintering. Rodrigues et al. (2010)

#### Tadorna ferruginea

(Pallas, 1764)

##### Ecological interactions

###### Native status

Palearctic

##### Distribution

SMG

##### Notes

Occasional Migrant. Rodrigues et al. (2010)

#### Tadorna tadorna

(Linnaeus, 1758)

##### Ecological interactions

###### Native status

Palearctic

##### Distribution

FLO; TER*; SMG

##### Notes

Occasional Migrant; Occasional Wintering. Rodrigues et al. (2010)

#### Galliformes

#### Phasianidae

#### Alectoris rufa hispanica

(Seoane, 1894)

##### Ecological interactions

###### Native status

Palearctic

###### Conservation status

A-IIA

##### Distribution

PIC (Breeder); GRA*; TER (Breeder); SMG*; SMR (Breeder)

##### Notes

Introduced. Rodrigues et al. (2010)

#### Coturnix coturnix conturbans

Hartert, 1917

##### Ecological interactions

###### Native status

Palearctic

###### Conservation status

A-IIB

##### Distribution

COR (Breeder); FLO (Breeder); FAI (Breeder); PIC (Breeder); GRA (Breeder); SJG (Breeder); TER (Breeder); SMG (Breeder); SMR (Breeder)

##### Notes

Native. Rodrigues et al. (2010)

#### Gaviiformes

#### Gaviidae

#### Gavia arctica

(Linnaeus, 1758)

##### Ecological interactions

###### Native status

Palearctic

##### Distribution

SMR

##### Notes

Occasional Migrant. New Azores Record

#### Gavia immer

(Brünnich, 1764)

##### Ecological interactions

###### Native status

Holarctic

##### Distribution

COR*; FLO; FAI; PIC; GRA; TER; SMG; SMR

##### Notes

Regular Wintering. Rodrigues et al. (2010)

#### Gavia stellata

(Pontoppidan, 1763)

##### Ecological interactions

###### Native status

Holarctic

##### Distribution

GRA; SMG

##### Notes

Occasional Wintering. Rodrigues et al. (2010)

#### Procellariiformes

#### Diomedeidae

#### Thalassarche melanophris

(Temminck, 1828)

##### Ecological interactions

###### Native status

Sub-Antarctic

##### Distribution

FAI; PIC

##### Notes

Occasional Migrant. Rodrigues et al. (2010)

#### Procellariidae

#### Bulweria bulwerii

(Jardine & Selby, 1828)

##### Ecological interactions

###### Native status

Pantropical

###### Conservation status

P; A-I; B-II; T100

##### Distribution

FLO*; FAI*; PIC*; GRA*; SMG*; SMR (Breeder)

##### Notes

Native. Rodrigues et al. (2010)

#### Calonectris borealis

(Cory, 1881)

##### Ecological interactions

###### Native status

Palearctic

###### Conservation status

P; A-I; B-II; T100

##### Distribution

COR (Breeder); FLO (Breeder); FAI (Breeder); PIC (Breeder); GRA (Breeder); SJG (Breeder); TER (Breeder); SMG (Breeder); SMR (Breeder)

##### Notes

Native. Rodrigues et al. (2010)

#### Fulmarus glacialis

(Linnaeus, 1761)

##### Ecological interactions

###### Native status

Holarctic

##### Distribution

COR; FLO; FAI; PIC; GRA*; TER; SMG; SMR

##### Notes

Occasional Migrant. Rodrigues et al. (2010)

#### Pterodroma arminjoniana

(Giglioli & Salvadori, 1868)

##### Ecological interactions

###### Native status

Pantropical

##### Distribution

COR; FLO*; FAI; PIC; GRA*

##### Notes

Occasional Migrant. Rodrigues et al. (2010)

#### Pterodroma cahow

(Nichols & Mowbray, 1916)

##### Ecological interactions

###### Native status

Nearctic

##### Distribution

SMR

##### Notes

Occasional and Non-Breeding. Rodrigues et al. (2010)

#### Pterodroma deserta

Mathews, 1934

##### Ecological interactions

###### Native status

Palearctic

##### Distribution

GRA

##### Notes

Occasional Migrant. New Azores Record

#### Pterodroma sp. (deserta or feae)

(Mathews, 1934) (Salvadori, 1899)

##### Ecological interactions

###### Native status

Palearctic

###### Conservation status

P; A-I; B-II

##### Distribution

COR; FLO; FAI; PIC; TER; SMG; SMR

##### Notes

Occasional Migrant. Rodrigues et al. (2010)

#### Pterodroma hasitata

(Kuhl, 1820)

##### Ecological interactions

###### Native status

Nearctic

##### Distribution

FAI*; PIC; GRA

##### Notes

Occasional Migrant. Rodrigues et al. (2010)

#### Pterodroma madeira

Mathews, 1934

##### Ecological interactions

###### Native status

Palearctic

##### Distribution

GRA

##### Notes

Occasional Migrant. New Azores Record

#### Pterodroma neglecta

(Schlegel, 1863)

##### Ecological interactions

###### Native status

Pantropical

##### Distribution

FAI

##### Notes

Occasional Migrant. Rodrigues et al. (2010)

#### Puffinus baroli

(Bonaparte, 1857)

##### Ecological interactions

###### Native status

Palearctic

###### Conservation status

P; A-I; B-II; O; T100

##### Distribution

COR (Breeder); FLO (Breeder); FAI (Breeder); PIC (Breeder); GRA (Breeder); SJG (Breeder); TER*; SMG (Breeder); SMR (Breeder)

##### Notes

Macaronesian Endemic. Rodrigues et al. (2010)

#### Puffinus gravis

(O'Reilly, 1818)

##### Ecological interactions

###### Native status

Southern Atlantic

###### Conservation status

A

##### Distribution

COR; FLO; FAI; PIC; GRA; SJG; TER; SMG; SMR

##### Notes

Regular Migrant. Rodrigues et al. (2010)

#### Puffinus griseus

(Gmelin, 1789)

##### Ecological interactions

###### Native status

Southern hemisphere

###### Conservation status

A

##### Distribution

COR; FLO; FAI; PIC; GRA; SJG; TER; SMG; SMR

##### Notes

Regular Migrant. Rodrigues et al. (2010)

#### Puffinus mauretanicus

Lowe, 1921

##### Ecological interactions

###### Native status

Palearctic

##### Distribution

FLO; FAI; SMG

##### Notes

Occasional Migrant. Rodrigues et al. (2010)

#### Puffinus puffinus

(Brünnich, 1764)

##### Ecological interactions

###### Native status

Holarctic

###### Conservation status

P; A; B-II

##### Distribution

COR (Breeder); FLO (Breeder); FAI*; PIC*; GRA*; SJG*; TER*; SMG (Breeder); SMR (Breeder)

##### Notes

Native. Rodrigues et al. (2010)

#### Hydrobatidae

#### Oceanites oceanicus

(Kuhl, 1820)

##### Ecological interactions

###### Native status

Sub-Antarctic and Antarctic

##### Distribution

COR*; FLO; FAI*; PIC; GRA*; TER*; SMG; SMR*

##### Notes

Regular Migrant. Rodrigues et al. (2010)

#### Oceanodroma castro

(Harcourt, 1851)

##### Ecological interactions

###### Native status

Pantropical

###### Conservation status

P; A-I; T100

##### Distribution

COR*; GRA (Breeder); SJG*; SMG*; SMR (Breeder)

##### Notes

Native. Rodrigues et al. (2010)

#### Oceanodroma leucorhoa

(Vieillot, 1818)

##### Ecological interactions

###### Native status

Holarctic

##### Distribution

COR*; FLO; FAI; PIC; GRA; TER; SMG; SMR

##### Notes

Regular Migrant. Rodrigues et al. (2010)

#### Oceanodroma monorhis

(Swinhoe, 1867)

##### Ecological interactions

###### Native status

Sub-tropical Pacific

##### Distribution

GRA

##### Notes

Occasional Migrant. New Azores Record

#### Oceanodroma monteiroi

Bolton et al., 2008

##### Ecological interactions

###### Native status

Palearctic

###### Conservation status

P; A-I (x)

##### Distribution

COR*; GRA (Breeder); SJG*

##### Notes

Azores Endemic. Rodrigues et al. (2010)

#### Pelagodroma marina

(Latham, 1790)

##### Ecological interactions

###### Native status

Palearctic

###### Conservation status

A

##### Distribution

FLO; FAI*; PIC; SMR

##### Notes

Occasional Migrant. Rodrigues et al. (2010)

#### Podicipediformes

#### Podicipedidae

#### Podiceps auritus

(Linnaeus, 1758)

##### Ecological interactions

###### Native status

Holarctic

##### Distribution

FLO; TER; SMG; SMR*

##### Notes

Occasional Wintering. Rodrigues et al. (2010)

#### Podiceps cristatus

(Linnaeus, 1758)

##### Ecological interactions

###### Native status

Palearctic

##### Distribution

SMG

##### Notes

Occasional Wintering. Rodrigues et al. (2010)

#### Podiceps grisegena

(Boddaert, 1783)

##### Ecological interactions

###### Native status

Holarctic

##### Distribution

FAI

##### Notes

Occasional Wintering. New Azores Record

#### Podiceps nigricollis

Brehm, 1831

##### Ecological interactions

###### Native status

Holarctic

##### Distribution

FAI; TER; SMG

##### Notes

Occasional Wintering. Rodrigues et al. (2010)

#### Podilymbus podiceps

(Linnaeus, 1758)

##### Ecological interactions

###### Native status

Nearctic

##### Distribution

COR; FLO; PIC; TER; SMG; SMR*

##### Notes

Occasional Wintering. Rodrigues et al. (2010)

#### Tachybaptus ruficollis

(Pallas, 1764)

##### Ecological interactions

###### Native status

Palearctic

##### Distribution

FLO; TER*; SMG

##### Notes

Occasional Wintering. Rodrigues et al. (2010)

#### Phoenicopteriformes

#### Phoenicopteridae

#### Phoenicopterus roseus

Pallas, 1811

##### Ecological interactions

###### Native status

Palearctic

##### Distribution

SMG

##### Notes

Occasional Migrant. Rodrigues et al. (2010)

#### Phaethontiformes

#### Phaethontidae

#### Phaethon aethereus mesonauta

Peters, 1930

##### Ecological interactions

###### Native status

Pantropical

###### Conservation status

A; AEWA

##### Distribution

FAI; PIC; GRA (Occasional Breeder); SMG*

##### Notes

Occasional Migrant. Rodrigues et al. (2010)

#### Phaethon lepturus

Daudin, 1802

##### Ecological interactions

###### Native status

Pantropical

##### Distribution

COR*; FLO; FAI*; SJG*; TER*; SMG*

##### Notes

Occasional Migrant. Rodrigues et al. (2010)

#### Ciconiiformes

#### Ciconiidae

#### Ciconia ciconia

(Linnaeus, 1758)

##### Ecological interactions

###### Native status

Palearctic

##### Distribution

PIC*; TER

##### Notes

Occasional Migrant. Rodrigues et al. (2010)

#### Ciconia nigra

(Linnaeus, 1758)

##### Ecological interactions

###### Native status

Palearctic

##### Distribution

SMG

##### Notes

Occasional Migrant. Rodrigues et al. (2010)

#### Pelecaniformes

#### Threskiornithidae

#### Geronticus eremita

(Linnaeus, 1758)

##### Ecological interactions

###### Native status

Palearctic

##### Distribution

SMG

##### Notes

Occasional Migrant. Rodrigues et al. (2010)

#### Platalea leucorodia

Linnaeus, 1758

##### Ecological interactions

###### Native status

Palearctic

##### Distribution

COR; FLO; PIC; TER; SMG; SMR

##### Notes

Occasional Migrant; Occasional Wintering. Rodrigues et al. (2010)

#### Plegadis falcinellus

(Linnaeus, 1766)

##### Ecological interactions

###### Native status

Holarctic

##### Distribution

COR*; FLO*; FAI*; PIC*; GRA*; SJG*; TER; SMG; SMR

##### Notes

Occasional Migrant. Rodrigues et al. (2010)

#### Ardeidae

#### Ardea alba alba

Linnaeus, 1758

##### Ecological interactions

###### Native status

Palearctic

##### Distribution

SMR

##### Notes

Occasional Migrant. Rodrigues et al. (2010)

#### Ardea alba egretta

Gmelin, 1789

##### Ecological interactions

###### Native status

Nearctic

##### Distribution

COR; FLO; FAI; PIC; GRA*; SJG; TER; SMG; SMR

##### Notes

Regular Migrant. Rodrigues et al. (2010)

#### Ardea cinerea

Linnaeus, 1758

##### Ecological interactions

###### Native status

Palearctic

###### Conservation status

A; AEWA

##### Distribution

COR; FLO; FAI; PIC; GRA; SJG; TER; SMG; SMR

##### Notes

Regular Migrant; Regular Wintering. Rodrigues et al. (2010)

#### Ardea herodias

Linnaeus, 1758

##### Ecological interactions

###### Native status

Nearctic

##### Distribution

COR; FLO; FAI; PIC; SJG; TER; SMG

##### Notes

Occasional Migrant; Occasional Wintering. Rodrigues et al. (2010)

#### Ardea purpurea

Linnaeus, 1766

##### Ecological interactions

###### Native status

Palearctic

##### Distribution

COR*; PIC*; TER; SMG; SMR*

##### Notes

Occasional Migrant. Rodrigues et al. (2010)

#### Ardeola ralloides

(Scopoli, 1769)

##### Ecological interactions

###### Native status

Palearctic

##### Distribution

COR*; FAI*; PIC; TER; SMG; SMR

##### Notes

Occasional Migrant. Rodrigues et al. (2010)

#### Botaurus lentiginosus

(Rackett, 1813)

##### Ecological interactions

###### Native status

Nearctic

##### Distribution

COR*; FLO; PIC*; GRA*; SJG; TER; SMG; SMR

##### Notes

Occasional Migrant. Rodrigues et al. (2010)

#### Botaurus stellaris

(Linnaeus, 1758)

##### Ecological interactions

###### Native status

Palearctic

##### Distribution

TER; SMG

##### Notes

Occasional Migrant. Rodrigues et al. (2010)

#### Bubulcus ibis

(Linnaeus, 1758)

##### Ecological interactions

###### Native status

Palearctic

###### Conservation status

A; AEWA

##### Distribution

COR; FLO; FAI; PIC; GRA; SJG; TER; SMG; SMR

##### Notes

Regular Wintering. Rodrigues et al. (2010)

#### Butorides virescens

(Linnaeus, 1758)

##### Ecological interactions

###### Native status

Nearctic

##### Distribution

FLO; PIC; SJG; SMG

##### Notes

Occasional Migrant. Rodrigues et al. (2010)

#### Egretta caerulea

(Linnaeus, 1758)

##### Ecological interactions

###### Native status

Nearctic

##### Distribution

FLO; PIC; GRA*; SJG; SMG*

##### Notes

Occasional Migrant. Rodrigues et al. (2010)

#### Egretta garzetta

(Linnaeus, 1766)

##### Ecological interactions

###### Native status

Palearctic

###### Conservation status

P; A-I; B-II; AEWA

##### Distribution

COR; FLO; FAI; PIC; GRA; SJG; TER; SMG; SMR

##### Notes

Regular Migrant; Regular Wintering. Rodrigues et al. (2010)

#### Egretta gularis

(Bosc, 1792)

##### Ecological interactions

###### Native status

Palearctic

##### Distribution

SMG

##### Notes

Occasional Migrant. Rodrigues et al. (2010)

#### Egretta thula

(Molina, 1782)

##### Ecological interactions

###### Native status

Nearctic

##### Distribution

FLO; FAI; PIC*; GRA*; SJG; TER; SMG

##### Notes

Occasional Migrant; Occasional Wintering. Rodrigues et al. (2010)

#### Egretta tricolor

(Müller, 1776)

##### Ecological interactions

###### Native status

Nearctic

##### Distribution

PIC; SMG

##### Notes

Occasional Migrant. Rodrigues et al. (2010)

#### Ixobrychus exilis

(Gmelin, 1789)

##### Ecological interactions

###### Native status

Nearctic

##### Distribution

TER; SMG; SMR

##### Notes

Occasional Migrant. Rodrigues et al. (2010)

#### Ixobrychus minutus

(Linnaeus, 1766)

##### Ecological interactions

###### Native status

Palearctic

##### Distribution

FLO; PIC; GRA; SJG; TER; SMG

##### Notes

Occasional Migrant. Rodrigues et al. (2010)

#### Nyctanassa violacea

(Linnaeus, 1758)

##### Ecological interactions

###### Native status

Nearctic

##### Distribution

COR; PIC; TER; SMR

##### Notes

Occasional Wintering. New Azores Record

#### Nycticorax nycticorax

(Linnaeus, 1758)

##### Ecological interactions

###### Native status

Holarctic

##### Distribution

COR; FLO; FAI; PIC; GRA; TER; SMG; SMR

##### Notes

Occasional Migrant. Rodrigues et al. (2010)

#### Suliformes

#### Fregatidae

#### Fregata magnificens

Mathews, 1914

##### Ecological interactions

###### Native status

Sub-tropical and Tropical Atlantic; Tropical-eastern Pacific

##### Distribution

COR*; FLO*; PIC*; TER*; SMG

##### Notes

Occasional Migrant. Rodrigues et al. (2010)

#### Sulidae

#### Morus bassanus

(Linnaeus, 1758)

##### Ecological interactions

###### Native status

Holarctic

##### Distribution

COR; FLO; FAI; PIC; GRA; SJG; TER; SMG; SMR

##### Notes

Regular Migrant; Regular Wintering. Rodrigues et al. (2010)

#### Sula dactylatra

Lesson, 1831

##### Ecological interactions

###### Native status

Pantropical

##### Distribution

FAI; GRA*

##### Notes

Occasional Migrant. Rodrigues et al. (2010)

#### Sula leucogaster

(Boddaert, 1783)

##### Ecological interactions

###### Native status

Pantropical

##### Distribution

GRA*; SMG

##### Notes

Occasional Migrant; Occasional Wintering. Rodrigues et al. (2010)

#### Phalacrocoracidae

#### Phalacrocorax auritus

(Lesson, 1831)

##### Ecological interactions

###### Native status

Nearctic

##### Distribution

COR; FLO; FAI; PIC; GRA*; TER; SMG; SMR

##### Notes

Occasional Wintering. Rodrigues et al. (2010)

#### Phalacrocorax carbo

(Linnaeus, 1758)

##### Ecological interactions

###### Native status

Holarctic

##### Distribution

COR; PIC*; GRA*; TER; SMG; SMR

##### Notes

Occasional Wintering. Rodrigues et al. (2010)

#### Accipitriformes

#### Pandiondidae

#### Pandion haliaetus

(Linnaeus, 1758)

##### Ecological interactions

###### Native status

Holarctic

##### Distribution

COR*; FLO; FAI; PIC; TER; SMG

##### Notes

Occasional Migrant. Rodrigues et al. (2010)

#### Accipitridae

#### Buteo buteo rothschildi

(Swann, 1919)

##### Ecological interactions

###### Native status

Palearctic

###### Conservation status

P; A; B-II; T100

##### Distribution

COR; FLO; FAI (Breeder); PIC (Breeder); GRA (Breeder); SJG (Breeder); TER (Breeder); SMG (Breeder); SMR (Breeder)

##### Notes

Azores Endemic. Rodrigues et al. (2010)

#### Buteo buteo buteo

(Linnaeus, 1758)

##### Ecological interactions

###### Native status

Palearctic

##### Distribution

GRA; TER

##### Notes

Occasional Migrant. New Azores Record

#### Buteo lagopus

(Pontoppidan, 1763)

##### Ecological interactions

###### Native status

Holarctic

##### Distribution

COR; FAI; PIC*; GRA*; TER

##### Notes

Occasional Migrant. Rodrigues et al. (2010)

#### Circus hudsonius

(Linnaeus, 1766)

##### Ecological interactions

###### Native status

Nearctic

##### Distribution

COR*; FLO; TER*

##### Notes

Occasional Migrant. Rodrigues et al. (2010)

#### Circus aeruginosus

(Linnaeus, 1758)

##### Ecological interactions

###### Native status

Palearctic

##### Distribution

FLO; TER; SMG; SMR*

##### Notes

Occasional Migrant. Rodrigues et al. (2010)

#### Circus cyaneus

(Linnaeus, 1766)

##### Ecological interactions

###### Native status

Palearctic

##### Distribution

COR; FLO; PIC*; TER; SMR

##### Notes

Occasional Migrant; Occasional Wintering. Rodrigues et al. (2010)

#### Circus pygargus

(Linnaeus, 1758)

##### Ecological interactions

###### Native status

Palearctic

##### Distribution

TER

##### Notes

Occasional Migrant. Rodrigues et al. (2010)

#### Elanoides forficatus

(Linnaeus, 1758)

##### Ecological interactions

###### Native status

Nearctic

##### Distribution

FLO; SMG

##### Notes

Occasional Migrant. Rodrigues et al. (2010)

#### Milvus migrans

(Boddaert, 1783)

##### Ecological interactions

###### Native status

Palearctic

##### Distribution

GRA; SMR

##### Notes

Occasional Migrant. New Azores Record

#### Milvus milvus

(Linnaeus, 1758)

##### Ecological interactions

###### Native status

Palearctic

##### Distribution

COR; SMG

##### Notes

Occasional Migrant. Rodrigues et al. (2010)

#### Neophron percnopterus

(Linnaeus, 1758)

##### Ecological interactions

###### Native status

Palearctic

##### Distribution

SMG

##### Notes

Occasional Migrant. Rodrigues et al. (2010)

#### Gruiformes

#### Rallidae

#### Crex crex

(Linnaeus, 1758)

##### Ecological interactions

###### Native status

Palearctic

##### Distribution

COR; FAI; TER; SMG; SMR

##### Notes

Occasional Migrant. Rodrigues et al. (2010)

#### Fulica americana

Gmelin, 1789

##### Ecological interactions

###### Native status

Nearctic

##### Distribution

FLO; FAI*; PIC; GRA*; TER; SMG

##### Notes

Occasional Wintering. Rodrigues et al. (2010)

#### Fulica atra atra

Linnaeus, 1758

##### Ecological interactions

###### Native status

Palearctic

###### Conservation status

A-IIA; AEWA

##### Distribution

COR; FLO; FAI; PIC; GRA; SJG; TER (Breeder); SMG (Breeder); SMR

##### Notes

Regular Wintering. Rodrigues et al. (2010)

#### Gallinula chloropus chloropus

(Linnaeus, 1758)

##### Ecological interactions

###### Native status

Palearctic

###### Conservation status

A-IIB; AEWA

##### Distribution

COR*; FLO (Breeder); FAI*; PIC*; GRA*; SJG*; TER (Breeder); SMG (Breeder); SMR (Breeder)

##### Notes

Native. Rodrigues et al. (2010)

#### Porphyrio alleni

Thomson, 1842

##### Ecological interactions

###### Native status

Palearctic

##### Distribution

GRA*; TER*; SMG; SMR

##### Notes

Occasional Migrant. Rodrigues et al. (2010)

#### Porphyrio martinicus

(Linnaeus, 1766)

##### Ecological interactions

###### Native status

Nearctic

##### Distribution

FLO; FAI*; GRA*; SMG

##### Notes

Occasional Migrant; Occasional Wintering. Rodrigues et al. (2010)

#### Porzana carolina

(Linnaeus, 1758)

##### Ecological interactions

###### Native status

Nearctic

##### Distribution

FLO*; SJG; SMR

##### Notes

Occasional Migrant. Rodrigues et al. (2010)

#### Porzana parva

(Scopoli, 1769)

##### Ecological interactions

###### Native status

Palearctic

##### Distribution

FAI; SMG

##### Notes

Occasional Migrant. Rodrigues et al. (2010)

#### Porzana porzana

(Linnaeus, 1766)

##### Ecological interactions

###### Native status

Palearctic

##### Distribution

COR; FLO*; PIC*; TER; SMG; SMR

##### Notes

Occasional Migrant. Rodrigues et al. (2010)

#### Porzana pusilla

(Pallas, 1776)

##### Ecological interactions

###### Native status

Palearctic

##### Distribution

SMG

##### Notes

Occasional Migrant. Rodrigues et al. (2010)

#### Rallus aquaticus

Linnaeus, 1758

##### Ecological interactions

###### Native status

Palearctic

##### Distribution

TER; SMG

##### Notes

Occasional Migrant. Rodrigues et al. (2010)

#### Gruidae

#### Grus canadensis

(Linnaeus, 1758)

##### Ecological interactions

###### Native status

Nearctic

##### Distribution

FLO

##### Notes

Occasional Migrant. Rodrigues et al. (2010)

#### Grus grus

(Linnaeus, 1758)

##### Ecological interactions

###### Native status

Palearctic

##### Distribution

SMG

##### Notes

Occasional Migrant. Rodrigues et al. (2010)

#### Charadriiformes

#### Burhinidae

#### Burhinus oedicnemus

(Linnaeus, 1758)

##### Ecological interactions

###### Native status

Palearctic

##### Distribution

SMG

##### Notes

Occasional Migrant. Rodrigues et al. (2010)

#### Haematopodidae

#### Haematopus ostralegus

Linnaeus, 1758

##### Ecological interactions

###### Native status

Palearctic

##### Distribution

FAI; SJG; TER; SMG; SMR

##### Notes

Occasional Migrant. Rodrigues et al. (2010)

#### Recurvirostridae

#### Himantopus himantopus

(Linnaeus, 1758)

##### Ecological interactions

###### Native status

Palearctic

##### Distribution

PIC; TER; SMG; SMR

##### Notes

Occasional Migrant. Rodrigues et al. (2010)

#### Recurvirostra avosetta

Linnaeus, 1758

##### Ecological interactions

###### Native status

Palearctic

##### Distribution

SMG; SMR*

##### Notes

Occasional Migrant. Rodrigues et al. (2010)

#### Charadriidae

#### Charadrius alexandrinus

Linnaeus, 1758

##### Ecological interactions

###### Native status

Palearctic

###### Conservation status

P; A-I; B-II; AEWA

##### Distribution

COR; FLO; FAI; PIC; GRA (Breeder); SJG (Breeder); TER (Breeder); SMG (Breeder); SMR (Breeder)

##### Notes

Regular Wintering. Rodrigues et al. (2010)

#### Charadrius asiaticus

Pallas, 1773

##### Ecological interactions

###### Native status

Palearctic

##### Distribution

COR

##### Notes

Occasional Migrant. New Azores Record

#### Charadrius dubius

Scopoli, 1786

##### Ecological interactions

###### Native status

Palearctic

###### Conservation status

A; B-II; AEWA

##### Distribution

COR; FLO; TER; SMG; SMR*

##### Notes

Occasional Migrant. Rodrigues et al. (2010)

#### Charadrius hiaticula

Linnaeus, 1758

##### Ecological interactions

###### Native status

Holarctic

###### Conservation status

A; B-II; AEWA

##### Distribution

COR; FLO; FAI; PIC; GRA; SJG; TER; SMG; SMR

##### Notes

Regular Migrant. Rodrigues et al. (2010)

#### Charadrius morinellus

Linnaeus, 1758

##### Ecological interactions

###### Native status

Palearctic

##### Distribution

COR; FAI; SMR

##### Notes

Occasional Migrant. Rodrigues et al. (2010)

#### Charadrius semipalmatus

Bonaparte, 1825

##### Ecological interactions

###### Native status

Nearctic

###### Conservation status

A

##### Distribution

COR; FLO; FAI; PIC; TER; SMG; SMR

##### Notes

Regular Migrant; Regular Wintering. Rodrigues et al. (2010)

#### Charadrius vociferus

Linnaeus, 1758

##### Ecological interactions

###### Native status

Nearctic

##### Distribution

COR; FLO; FAI; PIC*; TER; SMG; SMR* (Occasional Breeder)

##### Notes

Occasional Migrant. Rodrigues et al. (2010)

#### Pluvialis apricaria

(Linnaeus, 1758)

##### Ecological interactions

###### Native status

Palearctic

##### Distribution

COR*; FLO; FAI; GRA; TER; SMG; SMR

##### Notes

Occasional Migrant. Rodrigues et al. (2010)

#### Pluvialis dominica

(Müller, 1776)

##### Ecological interactions

###### Native status

Nearctic

##### Distribution

COR; FLO; FAI; PIC; TER; SMG; SMR

##### Notes

Occasional Migrant. Rodrigues et al. (2010)

#### Pluvialis fulva

(Gmelin, 1789)

##### Ecological interactions

###### Native status

Holarctic

##### Distribution

TER; SMR*

##### Notes

Occasional Migrant. Rodrigues et al. (2010)

#### Pluvialis squatarola

(Linnaeus, 1758)

##### Ecological interactions

###### Native status

Holarctic

###### Conservation status

A-IIB; AEWA

##### Distribution

COR; FLO; FAI; PIC; GRA; SJG; TER; SMG; SMR

##### Notes

Regular Migrant; Regular Wintering. Rodrigues et al. (2010)

#### Vanellus vanellus

(Linnaeus, 1758)

##### Ecological interactions

###### Native status

Palearctic

###### Conservation status

A-IIB; AEWA

##### Distribution

COR; FLO; FAI; PIC; GRA; SJG; TER; SMG; SMR

##### Notes

Occasional Wintering. Rodrigues et al. (2010)

#### Scolopacidae

#### Actitis hypoleucos

(Linnaeus, 1758)

##### Ecological interactions

###### Native status

Palearctic

##### Distribution

COR; FLO; FAI; PIC; SJG; TER; SMG; SMR

##### Notes

Occasional Migrant. Rodrigues et al. (2010)

#### Actitis macularius

(Linnaeus, 1766)

##### Ecological interactions

###### Native status

Nearctic

##### Distribution

COR; FLO; FAI; PIC; GRA; SJG; TER; SMG; SMR

##### Notes

Regular Migrant. Rodrigues et al. (2010)

#### Arenaria interpres

(Linnaeus, 1758)

##### Ecological interactions

###### Native status

Holarctic

###### Conservation status

A; B-II; AEWA

##### Distribution

COR; FLO; FAI; PIC; GRA; SJG; TER; SMG; SMR

##### Notes

Regular Migrant; Regular Wintering. Rodrigues et al. (2010)

#### Bartramia longicauda

(Bechstein, 1812)

##### Ecological interactions

###### Native status

Nearctic

##### Distribution

COR; FLO; SMG

##### Notes

Occasional Migrant. Rodrigues et al. (2010)

#### Calidris acuminata

(Horsfield, 1821)

##### Ecological interactions

###### Native status

Nearctic

##### Distribution

TER

##### Notes

Occasional Migrant. Rodrigues et al. (2010)

#### Calidris alba

(Pallas, 1764)

##### Ecological interactions

###### Native status

Holarctic

###### Conservation status

A; B-II; AEWA

##### Distribution

COR; FLO; FAI; PIC; GRA; SJG; TER; SMG; SMR

##### Notes

Regular Migrant; Regular Wintering. Rodrigues et al. (2010)

#### Calidris alpina

(Linnaeus, 1758)

##### Ecological interactions

###### Native status

Holarctic

###### Conservation status

P; A-I; B-II; AEWA

##### Distribution

FLO; FAI; PIC; GRA; SJG; TER; SMG; SMR

##### Notes

Occasional Migrant. Rodrigues et al. (2010)

#### Calidris bairdii

(Coues, 1861)

##### Ecological interactions

###### Native status

Holarctic

##### Distribution

FLO*; SJG; TER; SMG

##### Notes

Occasional Migrant. Rodrigues et al. (2010)

#### Calidris canutus

(Linnaeus, 1758)

##### Ecological interactions

###### Native status

Holarctic

###### Conservation status

A-IIB; AEWA

##### Distribution

COR; FLO; FAI; PIC; GRA; SJG; TER; SMG; SMR

##### Notes

Regular Migrant. Rodrigues et al. (2010)

#### Calidris ferruginea

(Pontoppidan, 1763)

##### Ecological interactions

###### Native status

Palearctic

###### Conservation status

A; B-II; AEWA

##### Distribution

COR; FLO; FAI; PIC; TER; SMG; SMR

##### Notes

Occasional Migrant. Rodrigues et al. (2010)

#### Calidris fuscicollis

(Vieillot, 1819)

##### Ecological interactions

###### Native status

Nearctic

###### Conservation status

A

##### Distribution

COR; FLO; FAI; PIC; GRA*; SJG; TER; SMG; SMR*

##### Notes

Regular Migrante; Occasional Wintering. Rodrigues et al. (2010)

#### Calidris himantopus

(Bonaparte, 1826)

##### Ecological interactions

###### Native status

Nearctic

##### Distribution

PIC; SJG*; SMG

##### Notes

Occasional Migrant. Rodrigues et al. (2010)

#### Calidris maritima

(Brünnich, 1764)

##### Ecological interactions

###### Native status

Holarctic

##### Distribution

COR; FLO; FAI; PIC; GRA; TER; SMG

##### Notes

Occasional Migrant; Occasional Wintering. Rodrigues et al. (2010)

#### Calidris mauri

(Cabanis, 1857)

##### Ecological interactions

###### Native status

Nearctic

##### Distribution

COR*; PIC; TER; SMG; SMR

##### Notes

Occasional Migrant. Rodrigues et al. (2010)

#### Calidris melanotos

(Vieillot, 1819)

##### Ecological interactions

###### Native status

Holarctic

##### Distribution

COR; FLO; FAI; PIC; SJG; TER; SMG; SMR

##### Notes

Regular Migrant. Rodrigues et al. (2010)

#### Calidris minuta

(Leisler, 1812)

##### Ecological interactions

###### Native status

Palearctic

##### Distribution

COR; FLO; FAI; PIC; SJG; TER; SMG; SMR

##### Notes

Occasional Migrant; Occasional Wintering. Rodrigues et al. (2010)

#### Calidris minutilla

(Vieillot, 1819)

##### Ecological interactions

###### Native status

Nearctic

##### Distribution

COR; FLO; FAI; PIC; SJG; TER; SMG

##### Notes

Occasional Migrant; Occasional Wintering. Rodrigues et al. (2010)

#### Calidris pusilla

(Linnaeus, 1766)

##### Ecological interactions

###### Native status

Nearctic

##### Distribution

COR; FLO; FAI; PIC; GRA*; SJG; TER; SMG; SMR*

##### Notes

Regular Migrante; Occasional Wintering. Rodrigues et al. (2010)

#### Calidris temminckii

(Leisler, 1812)

##### Ecological interactions

###### Native status

Palearctic

##### Distribution

TER; SMG*

##### Notes

Occasional Migrant. Rodrigues et al. (2010)

#### Gallinago delicata

(Ord, 1825)

##### Ecological interactions

###### Native status

Nearctic

##### Distribution

COR; FLO; FAI*; PIC; GRA; SJG; TER; SMG; SMR*

##### Notes

Regular Migrant. Rodrigues et al. (2010)

#### Gallinago gallinago gallinago

(Linnaeus, 1758)

##### Ecological interactions

###### Native status

Palearctic

###### Conservation status

A-IIA; AEWA

##### Distribution

COR (Breeder); FLO (Breeder); FAI (Breeder); PIC (Breeder); GRA*; SJG (Breeder); TER (Breeder); SMG (Breeder); SMR*

##### Notes

Native. Rodrigues et al. (2010)

#### Gallinago media

(Latham, 1787)

##### Ecological interactions

###### Native status

Palearctic

##### Distribution

SMR

##### Notes

Occasional Migrant. New Azores Record

#### Limnodromus griseus

(Gmelin, 1789)

##### Ecological interactions

###### Native status

Nearctic

##### Distribution

COR; FLO; PIC; SJG; TER; SMG; SMR*

##### Notes

Occasional Migrant; Occasional Wintering. Rodrigues et al. (2010)

#### Limnodromus scolopaceus

(Say, 1822)

##### Ecological interactions

###### Native status

Nearctic

##### Distribution

COR; TER; SMG; SMR*

##### Notes

Occasional Migrant; Occasional Wintering. Rodrigues et al. (2010)

#### Limosa haemastica

(Linnaeus, 1758)

##### Ecological interactions

###### Native status

Nearctic

##### Distribution

TER

##### Notes

Occasional Migrant. Rodrigues et al. (2010)

#### Limosa lapponica

(Linnaeus, 1758)

##### Ecological interactions

###### Native status

Palearctic

##### Distribution

COR; FLO*; FAI; PIC; GRA; SJG; TER; SMG; SMR

##### Notes

Regular Migrant. Rodrigues et al. (2010)

#### Limosa limosa

(Linnaeus, 1758)

##### Ecological interactions

###### Native status

Palearctic

###### Conservation status

A-IIB; AEWA

##### Distribution

SJG; TER; SMG

##### Notes

Regular Migrant. Rodrigues et al. (2010)

#### Lymnocryptes minimus

(Brünnich, 1764)

##### Ecological interactions

###### Native status

Palearctic

##### Distribution

COR; FAI; TER; SMG; SMR

##### Notes

Occasional Migrant. Rodrigues et al. (2010)

#### Numenius arquata

(Linnaeus, 1758)

##### Ecological interactions

###### Native status

Palearctic

##### Distribution

FLO*; PIC; GRA; TER; SMG; SMR

##### Notes

Occasional Migrant; Occasional Wintering. Rodrigues et al. (2010)

#### Numenius phaeopus hudsonicus

Latham, 1790

##### Ecological interactions

###### Native status

Nearctic

##### Distribution

COR; FLO; FAI*; PIC*; GRA; SJG*; TER; SMG; SMR*

##### Notes

Occasional Migrant. Rodrigues et al. (2010)

#### Numenius phaeopus phaeopus

(Linnaeus, 1758)

##### Ecological interactions

###### Native status

Palearctic

###### Conservation status

A-IIB; AEWA

##### Distribution

COR; FLO; FAI; PIC; GRA; SJG; TER; SMG; SMR

##### Notes

Regular Migrant; Regular Wintering. Rodrigues et al. (2010)

#### Numenius tenuirostris

Vieillot, 1817

##### Ecological interactions

###### Native status

Palearctic

##### Distribution

SMG

##### Notes

Occasional Migrant. Rodrigues et al. (2010)

#### Phalaropus fulicarius

(Linnaeus, 1758)

##### Ecological interactions

###### Native status

Holarctic

##### Distribution

COR; FLO; FAI; PIC; GRA; SJG; TER; SMG; SMR

##### Notes

Occasional Migrant. Rodrigues et al. (2010)

#### Phalaropus lobatus

(Linnaeus, 1758)

##### Ecological interactions

###### Native status

Holarctic

##### Distribution

TER*; SMG

##### Notes

Occasional Migrant. Rodrigues et al. (2010)

#### Phalaropus tricolor

(Vieillot, 1819)

##### Ecological interactions

###### Native status

Nearctic

##### Distribution

COR; PIC; TER; SMG

##### Notes

Occasional Migrant. Rodrigues et al. (2010)

#### Philomachus pugnax

(Linnaeus, 1758)

##### Ecological interactions

###### Native status

Palearctic

###### Conservation status

P; A-I;A-IIB; AEWA

##### Distribution

COR; FLO; FAI; PIC; GRA*; SJG; TER; SMG; SMR

##### Notes

Occasional Migrant. Rodrigues et al. (2010)

#### Scolopax rusticola

Linnaeus, 1758

##### Ecological interactions

###### Native status

Palearctic

###### Conservation status

A-IIA; AEWA

##### Distribution

COR (Breeder); FLO (Breeder); FAI (Breeder); PIC (Breeder); GRA*; SJG (Breeder); TER (Breeder); SMG (Breeder); SMR*

##### Notes

Native. Rodrigues et al. (2010)

#### Tringa erythropus

(Pallas, 1764)

##### Ecological interactions

###### Native status

Palearctic

##### Distribution

FAI; TER; SMG

##### Notes

Occasional Migrant. Rodrigues et al. (2010)

#### Tringa flavipes

(Gmelin, 1789)

##### Ecological interactions

###### Native status

Nearctic

##### Distribution

COR; FLO; FAI; PIC; GRA; SJG; TER; SMG; SMR*

##### Notes

Regular Migrant. Rodrigues et al. (2010)

#### Tringa glareola

Linnaeus, 1758

##### Ecological interactions

###### Native status

Palearctic

##### Distribution

FLO; PIC*; SJG; TER; SMG; SMR

##### Notes

Occasional Migrant. Rodrigues et al. (2010)

#### Tringa melanoleuca

(Gmelin, 1789)

##### Ecological interactions

###### Native status

Nearctic

##### Distribution

COR; FLO; PIC; TER; SMG

##### Notes

Occasional Migrant. Rodrigues et al. (2010)

#### Tringa nebularia

(Gunnerus, 1767)

##### Ecological interactions

###### Native status

Palearctic

###### Conservation status

A-IIB; AEWA

##### Distribution

COR; FLO; FAI; PIC; GRA; SJG; TER; SMG; SMR

##### Notes

Regular Migrant. Rodrigues et al. (2010)

#### Tringa ochropus

Linnaeus, 1758

##### Ecological interactions

###### Native status

Palearctic

##### Distribution

FLO; PIC; TER; SMG; SMR

##### Notes

Occasional Migrant. Rodrigues et al. (2010)

#### Tringa semipalmata

(Gmelin, 1789)

##### Ecological interactions

###### Native status

Nearctic

##### Distribution

SJG; TER; SMG

##### Notes

Occasional Migrant. Rodrigues et al. (2010)

#### Tringa solitaria

Wilson, 1813

##### Ecological interactions

###### Native status

Nearctic

##### Distribution

COR*; FLO; GRA*; TER; SMG; SMR

##### Notes

Occasional Migrant. Rodrigues et al. (2010)

#### Tringa stagnatilis

(Bechstein, 1803)

##### Ecological interactions

###### Native status

Palearctic

##### Distribution

FLO; PIC*; TER; SMG; SMR

##### Notes

Occasional Migrant. Rodrigues et al. (2010)

#### Tringa totanus

(Linnaeus, 1758)

##### Ecological interactions

###### Native status

Palearctic

##### Distribution

FLO; SJG; TER; SMG; SMR

##### Notes

Occasional Migrant; Occasional Wintering. Rodrigues et al. (2010)

#### Tryngites subruficollis

(Vieillot, 1819)

##### Ecological interactions

###### Native status

Nearctic

##### Distribution

COR; FLO; FAI; PIC; TER; SMG; SMR*

##### Notes

Occasional Migrant. Rodrigues et al. (2010)

#### Xenus cinereus

(Güldenstädt, 1775)

##### Ecological interactions

###### Native status

Holarctic

##### Distribution

TER

##### Notes

Occasional Wintering. New Azores Record

#### Laridae

#### Anous stolidus

(Linnaeus, 1758)

##### Ecological interactions

###### Native status

Pantropical

##### Distribution

FLO

##### Notes

Occasional Migrant. Rodrigues et al. (2010)

#### Chlidonias niger niger

(Linnaeus, 1758)

##### Ecological interactions

###### Native status

Palearctic

##### Distribution

FLO; TER; SMG*

##### Notes

Occasional Migrant. Rodrigues et al. (2010)

#### Chlidonias niger surinamensis

(Gmelin, 1789)

##### Ecological interactions

###### Native status

Nearctic

##### Distribution

COR; TER; SMG*

##### Notes

Occasional Migrant. Rodrigues et al. (2010)

#### Chlidonias hybrida

(Pallas, 1811)

##### Ecological interactions

###### Native status

Palearctic

##### Distribution

TER; SMG; SMR

##### Notes

Occasional Migrant. Rodrigues et al. (2010)

#### Chlidonias leucopterus

(Temminck, 1815)

##### Ecological interactions

###### Native status

Palearctic

##### Distribution

TER; SMG; SMR*

##### Notes

Occasional Migrant. Rodrigues et al. (2010)

#### Chroicocephalus philadelphia

(Ord, 1815)

##### Ecological interactions

###### Native status

Nearctic

##### Distribution

FAI; PIC; TER; SMG

##### Notes

Occasional Migrant. Rodrigues et al. (2010)

#### Chroicocephalus ridibundus

(Linnaeus, 1766)

##### Ecological interactions

###### Native status

Palearctic

###### Conservation status

A-IIB; AEWA

##### Distribution

COR; FLO; FAI; PIC; GRA; SJG; TER; SMG; SMR

##### Notes

Regular Migrant; Regular Wintering. Rodrigues et al. (2010)

#### Gelochelidon nilotica

(Gmelin, 1789)

##### Ecological interactions

###### Native status

Holarctic

##### Distribution

PIC; TER; SMG

##### Notes

Occasional Migrant. Rodrigues et al. (2010)

#### Hydrocoloeus minutus

(Pallas, 1776)

##### Ecological interactions

###### Native status

Palearctic

##### Distribution

FAI; SMG; SMR

##### Notes

Occasional Migrant. Rodrigues et al. (2010)

#### Hydroprogne caspia

(Pallas, 1770)

##### Ecological interactions

###### Native status

Holarctic

##### Distribution

FLO; TER; SMG*

##### Notes

Occasional Migrant. Rodrigues et al. (2010)

#### Ichthyaetus audouinii

(Payraudeau, 1826)

##### Ecological interactions

###### Native status

Palearctic

##### Distribution

SMG

##### Notes

Occasional Migrant. Rodrigues et al. (2010)

#### Ichthyaetus melanocephalus

(Temminck, 1820)

##### Ecological interactions

###### Native status

Palearctic

##### Distribution

FAI; PIC*; TER; SMG

##### Notes

Occasional Migrant; Occasional Wintering. Rodrigues et al. (2010)

#### Larus argentatus

Pontoppidan, 1763

##### Ecological interactions

###### Native status

Palearctic

##### Distribution

FLO; FAI; TER; SMG

##### Notes

Occasional Migrant. Rodrigues et al. (2010)

#### Larus canus brachyrhynchus

Richardson, 1831

##### Ecological interactions

###### Native status

Nearctic

##### Distribution

TER

##### Notes

Occasional Wintering. Rodrigues et al. (2010)

#### Larus canus canus

(Linnaeus, 1758)

##### Ecological interactions

###### Native status

Palearctic

##### Distribution

COR; FLO; PIC; TER; SMG

##### Notes

Occasional Wintering. Rodrigues et al. (2010)

#### Larus delawarensis

Ord, 1815

##### Ecological interactions

###### Native status

Nearctic

###### Conservation status

A

##### Distribution

COR; FLO; FAI; PIC; GRA; SJG; TER; SMG; SMR

##### Notes

Regular Wintering. Rodrigues et al. (2010)

#### Larus fuscus

Linnaeus, 1758

##### Ecological interactions

###### Native status

Palearctic

##### Distribution

COR; FLO; FAI; PIC; GRA; SJG; TER; SMG; SMR

##### Notes

Regular Migrant; Regular Wintering. Rodrigues et al. (2010)

#### Larus glaucoides glaucoides

Meyer, 1822

##### Ecological interactions

###### Native status

Holarctic

##### Distribution

COR; FLO; FAI; PIC; GRA; TER; SMG

##### Notes

Occasional Wintering. Rodrigues et al. (2010)

#### Larus glaucoides kumlieni

Brewster, 1883

##### Ecological interactions

###### Native status

Nearctic

##### Distribution

FLO; TER*; SMG; SMR*

##### Notes

Occasional Wintering. Rodrigues et al. (2010)

#### Larus hyperboreus

Gunnerus, 1767

##### Ecological interactions

###### Native status

Holarctic

##### Distribution

COR; FLO; FAI; PIC; GRA; SJG; TER; SMG; SMR

##### Notes

Regular Wintering. Rodrigues et al. (2010)

#### Larus marinus

Linnaeus, 1758

##### Ecological interactions

###### Native status

Holarctic

###### Conservation status

A-IIB; AEWA

##### Distribution

COR; FLO; FAI; PIC; GRA; SJG; TER; SMG; SMR

##### Notes

Regular Wintering. Rodrigues et al. (2010)

#### Larus michahellis atlantis

Dwight, 1922

##### Ecological interactions

###### Native status

Palearctic

###### Conservation status

P; A-IIB; AEWA

##### Distribution

COR (Breeder); FLO (Breeder); FAI (Breeder); PIC (Breeder); GRA (Breeder); SJG (Breeder); TER (Breeder); SMG (Breeder); SMR (Breeder)

##### Notes

Azores Endemic. Rodrigues et al. (2010)

#### Larus michahellis michahellis

Naumann, 1840

##### Ecological interactions

###### Native status

Palearctic

##### Distribution

COR; FLO; FAI; PIC; GRA; SJG; TER; SMG; SMR

##### Notes

Occasional Migrant. Rodrigues et al. (2010)

#### Larus smithsonianus

Coues, 1862

##### Ecological interactions

###### Native status

Nearctic

##### Distribution

COR*; FLO; FAI; PIC; SJG*; TER; SMG

##### Notes

Occasional Wintering. Rodrigues et al. (2010)

#### Leucophaeus atricilla

(Linnaeus, 1758)

##### Ecological interactions

###### Native status

Nearctic

##### Distribution

COR; FLO; FAI; PIC; GRA; TER; SMG; SMR

##### Notes

Occasional Migrant; Occasional Wintering. Rodrigues et al. (2010)

#### Leucophaeus pipixcan

(Wagler, 1831)

##### Ecological interactions

###### Native status

Nearctic

##### Distribution

FLO; TER; SMG; SMR*

##### Notes

Occasional Migrant. Rodrigues et al. (2010)

#### Onychoprion anaethetus melanopterus

(Swainson, 1837)

##### Ecological interactions

###### Native status

Pantropical

##### Distribution

PIC; GRA (Occasional Breeder); SJG; TER; SMG; SMR

##### Notes

Occasional Migrant. Rodrigues et al. (2010)

#### Onychoprion fuscatus fuscatus

(Linnaeus, 1766)

##### Ecological interactions

###### Native status

Tropical Atlantic

###### Conservation status

A; AEWA

##### Distribution

FAI*; GRA (Breeder); SJG*; TER*; SMG*; SMR

##### Notes

Native. Rodrigues et al. (2010)

#### Rissa tridactyla

(Linnaeus, 1758)

##### Ecological interactions

###### Native status

Holarctic

###### Conservation status

A; AEWA

##### Distribution

COR; FLO; FAI; PIC; GRA; SJG; TER; SMG; SMR

##### Notes

Regular Migrant; Regular Wintering. Rodrigues et al. (2010)

#### Sterna dougallii dougallii

Montagu, 1813

##### Ecological interactions

###### Native status

Holarctic

###### Conservation status

P; A-I; B-II; AEWA; O; T100

##### Distribution

COR (Breeder); FLO (Breeder); FAI (Breeder); PIC (Breeder); GRA (Breeder); SJG (Breeder); TER (Breeder); SMG (Breeder); SMR (Breeder)

##### Notes

Native. Rodrigues et al. (2010)

#### Sterna forsteri

Nuttall, 1834

##### Ecological interactions

###### Native status

Nearctic

##### Distribution

COR; FLO; TER; SMG*

##### Notes

Occasional Migrant. Rodrigues et al. (2010)

#### Sterna hirundo hirundo

Linnaeus, 1758

##### Ecological interactions

###### Native status

Holarctic

###### Conservation status

P; A-I; B-II; AEWA; T100

##### Distribution

COR (Breeder); FLO (Breeder); FAI (Breeder); PIC (Breeder); GRA (Breeder); SJG (Breeder); TER (Breeder); SMG (Breeder); SMR (Breeder)

##### Notes

Native. Rodrigues et al. (2010)

#### Sterna paradisaea

Pontoppidan, 1763

##### Ecological interactions

###### Native status

Holarctic

##### Distribution

COR; FLO; FAI; TER; SMG; SMR*

##### Notes

Occasional Migrant. Rodrigues et al. (2010)

#### Sternula albifrons

(Pallas, 1764)

##### Ecological interactions

###### Native status

Palearctic

##### Distribution

FLO; SMG

##### Notes

Occasional Migrant. Rodrigues et al. (2010)

#### Thalasseus maximus

(Boddaert, 1783)

##### Ecological interactions

###### Native status

Holarctic

##### Distribution

FLO; GRA; TER

##### Notes

Occasional Migrant. Rodrigues et al. (2010)

#### Thalasseus sandvicensis

(Latham, 1787)

##### Ecological interactions

###### Native status

Palearctic

##### Distribution

COR; FLO; FAI; PIC; GRA; SJG; TER; SMG

##### Notes

Occasional Migrant. Rodrigues et al. (2010)

#### Xema sabini

(Sabine, 1819)

##### Ecological interactions

###### Native status

Holarctic

##### Distribution

COR*; FLO; FAI; PIC*; GRA; SMG; SMR

##### Notes

Occasional Migrant. Rodrigues et al. (2010)

#### Stercorariidae

#### Stercorarius longicaudus

Vieillot, 1819

##### Ecological interactions

###### Native status

Holarctic

##### Distribution

FAI; PIC; GRA*; SJG; TER; SMG; SMR

##### Notes

Occasional Migrant. Rodrigues et al. (2010)

#### Stercorarius maccormicki

Saunders, 1893

##### Ecological interactions

###### Native status

Antarctic

##### Distribution

FLO*; FAI*; GRA*; SMG

##### Notes

Occasional Migrant. Rodrigues et al. (2010)

#### Stercorarius parasiticus

(Linnaeus, 1758)

##### Ecological interactions

###### Native status

Holarctic

##### Distribution

COR; FLO; FAI; PIC; GRA; SJG; TER; SMG; SMR

##### Notes

Regular Migrant. Rodrigues et al. (2010)

#### Stercorarius pomarinus

(Temminck, 1815)

##### Ecological interactions

###### Native status

Holarctic

##### Distribution

COR; FLO; FAI; PIC; GRA; SJG*; TER; SMG; SMR

##### Notes

Regular Migrant. Rodrigues et al. (2010)

#### Stercorarius skua

(Brünnich, 1764)

##### Ecological interactions

###### Native status

Palearctic

##### Distribution

COR; FLO; FAI; PIC; GRA; SJG; TER; SMG; SMR

##### Notes

Regular Migrant. Rodrigues et al. (2010)

#### Alcidae

#### Alca torda

Linnaeus, 1758

##### Ecological interactions

###### Native status

Holarctic

##### Distribution

FAI; TER; SMG

##### Notes

Occasional Wintering. Rodrigues et al. (2010)

#### Alle alle

(Linnaeus, 1758)

##### Ecological interactions

###### Native status

Palearctic

##### Distribution

COR; FAI; PIC; GRA; SJG; TER; SMG

##### Notes

Occasional Wintering. Rodrigues et al. (2010)

#### Fratercula arctica

(Linnaeus, 1758)

##### Ecological interactions

###### Native status

Holarctic

##### Distribution

FLO; FAI; PIC; GRA*; TER; SMG; SMR

##### Notes

Occasional Wintering. Rodrigues et al. (2010)

#### Uria lomvia

(Linnaeus, 1758)

##### Ecological interactions

###### Native status

Holarctic

##### Distribution

FLO*; FAI; PIC*; TER; SMG

##### Notes

Occasional Wintering. Rodrigues et al. (2010)

#### Columbiformes

#### Columbidae

#### Columba livia (domestica)

Gmelin, 1789

##### Ecological interactions

###### Native status

Palearctic

###### Conservation status

A-IIA

##### Distribution

COR (Breeder); FLO (Breeder); FAI (Breeder); PIC (Breeder); GRA (Breeder); SJG (Breeder); TER (Breeder); SMG (Breeder); SMR (Breeder)

##### Notes

Introduced. Rodrigues et al. (2010)

#### Columba palumbus azorica

Hartert, 1905

##### Ecological interactions

###### Native status

Palearctic

###### Conservation status

P; A-I

##### Distribution

COR (Breeder); FLO (Breeder); FAI (Breeder); PIC (Breeder); GRA (Breeder); SJG (Breeder); TER (Breeder); SMG (Breeder); SMR (Breeder)

##### Notes

Azores Endemic. Rodrigues et al. (2010)

#### Streptopelia decaocto

(Frivaldszky, 1838)

##### Ecological interactions

###### Native status

Palearctic

##### Distribution

COR*; FAI*; PIC (Breeder); GRA*; TER (Breeder); SMG; SMR

##### Notes

Native. Rodrigues et al. (2010)

#### Streptopelia turtur

(Linnaeus, 1758)

##### Ecological interactions

###### Native status

Palearctic

##### Distribution

COR; FLO; TER; SMG; SMR*

##### Notes

Occasional Migrant. Rodrigues et al. (2010)

#### Zenaida macroura

(Linnaeus, 1758)

##### Ecological interactions

###### Native status

Nearctic

##### Distribution

COR; FLO

##### Notes

Occasional Migrant. Rodrigues et al. (2010)

#### Cuculiformes

#### Cuculidae

#### Coccyzus americanus

(Linnaeus, 1758)

##### Ecological interactions

###### Native status

Nearctic

##### Distribution

COR; FLO; FAI; SJG; TER; SMG; SMR

##### Notes

Occasional Migrant. Rodrigues et al. (2010)

#### Coccyzus erythropthalmus

(Wilson, 1811)

##### Ecological interactions

###### Native status

Nearctic

##### Distribution

COR*; FLO; SMG

##### Notes

Occasional Migrant. Rodrigues et al. (2010)

#### Cuculus canorus

Linnaeus, 1758

##### Ecological interactions

###### Native status

Palearctic

##### Distribution

PIC*; SJG; TER*; SMG; SMR

##### Notes

Occasional Migrant. Rodrigues et al. (2010)

#### Strigiformes

#### Tytonidae

#### Tyto alba

(Scopoli, 1769)

##### Ecological interactions

###### Native status

Holarctic

##### Distribution

SMG

##### Notes

Occasional Migrant. Rodrigues et al. (2010)

#### Strigidae

#### Asio flammeus

(Pontoppidan, 1763)

##### Ecological interactions

###### Native status

Holarctic

##### Distribution

COR; FLO; FAI; PIC; GRA; SJG*; TER; SMG; SMR

##### Notes

Occasional Migrant; Occasional Wintering. Rodrigues et al. (2010)

#### Asio otus otus

(Linnaeus, 1758)

##### Ecological interactions

###### Native status

Palearctic

###### Conservation status

A; B-II

##### Distribution

COR*; FLO*; FAI (Breeder); PIC (Breeder); GRA (Breeder); SJG (Breeder); TER (Breeder); SMG (Breeder); SMR*

##### Notes

Native. Rodrigues et al. (2010)

#### Bubo scandiacus

(Linnaeus, 1758)

##### Ecological interactions

###### Native status

Holarctic

##### Distribution

COR*; FLO; FAI; PIC*; SJG*

##### Notes

Occasional Wintering. Rodrigues et al. (2010)

#### Caprimulgiformes

#### Caprimulgidae

#### Caprimulgus europaeus

Linnaeus, 1758

##### Ecological interactions

###### Native status

Palearctic

##### Distribution

COR*; GRA*; TER; SMG

##### Notes

Occasional Migrant. Rodrigues et al. (2010)

#### Chordeiles minor

(Forster, 1771)

##### Ecological interactions

###### Native status

Nearctic

##### Distribution

COR; FLO; FAI; PIC; SJG; TER; SMG

##### Notes

Occasional Migrant. Rodrigues et al. (2010)

#### Apodiformes

#### Apodidae

#### Apus affinis

(Gray, 1830)

##### Ecological interactions

###### Native status

Afro-tropical

##### Distribution

SMR

##### Notes

Occasional Migrant. Rodrigues et al. (2010)

#### Apus apus

(Linnaeus, 1758)

##### Ecological interactions

###### Native status

Palearctic

##### Distribution

FLO; FAI; GRA; TER; SMG; SMR

##### Notes

Occasional Migrant. Rodrigues et al. (2010)

#### Apus pallidus

(Shelley, 1870)

##### Ecological interactions

###### Native status

Palearctic

##### Distribution

FLO*; SMG

##### Notes

Occasional Migrant. Rodrigues et al. (2010)

#### Chaetura pelagica

(Linnaeus, 1758)

##### Ecological interactions

###### Native status

Nearctic

##### Distribution

COR; FLO; FAI; TER; SMG

##### Notes

Occasional Migrant. Rodrigues et al. (2010)

#### Tachymarptis melba

(Linnaeus, 1758)

##### Ecological interactions

###### Native status

Palearctic

##### Distribution

COR; FAI*; TER*; SMG; SMR*

##### Notes

Occasional Migrant. Rodrigues et al. (2010)

#### Coraciiformes

#### Caoraciidae

#### Coracias garrulus

Linnaeus, 1758

##### Ecological interactions

###### Native status

Palearctic

##### Distribution

SMG; SMR

##### Notes

Occasional Migrant. Rodrigues et al. (2010)

#### Alcedinidae

#### Alcedo atthis

(Linnaeus, 1758)

##### Ecological interactions

###### Native status

Palearctic

##### Distribution

SMR

##### Notes

Occasional Migrant. Rodrigues et al. (2010)

#### Megaceryle alcyon

(Linnaeus, 1758)

##### Ecological interactions

###### Native status

Nearctic

##### Distribution

FLO; FAI; PIC; GRA; SJG; TER*; SMR*

##### Notes

Occasional Migrant; Occasional Wintering. Rodrigues et al. (2010)

#### Meropidae

#### Merops apiaster

Linnaeus, 1758

##### Ecological interactions

###### Native status

Palearctic

##### Distribution

SMG

##### Notes

Occasional Migrant. Rodrigues et al. (2010)

#### Bucerotiformes

#### Upupidae

#### Upupa epops

Linnaeus, 1758

##### Ecological interactions

###### Native status

Palearctic

##### Distribution

FLO; PIC*; SJG; TER; SMG; SMR

##### Notes

Occasional Migrant. Rodrigues et al. (2010)

#### Piciformes

#### Picidae

#### Colaptes auratus

(Linnaeus, 1758)

##### Ecological interactions

###### Native status

Nearctic

##### Distribution

COR; FAI

##### Notes

Occasional Migrant. New Azores Record

#### Sphyrapicus varius

(Linnaeus, 1766)

##### Ecological interactions

###### Native status

Nearctic

##### Distribution

COR

##### Notes

Occasional Migrant. Rodrigues et al. (2010)

#### Falconiformes

#### Falconidae

#### Falco amurensis

Radde, 1863

##### Ecological interactions

###### Native status

Palearctic

##### Distribution

PIC

##### Notes

Occasional Migrant. New Azores Record

#### Falco columbarius

Linnaeus, 1758

##### Ecological interactions

###### Native status

Holarctic

##### Distribution

COR; FLO; TER; SMG; SMR*

##### Notes

Occasional Migrant. Rodrigues et al. (2010)

#### Falco eleonorae

Géné, 1839

##### Ecological interactions

###### Native status

Palearctic

##### Distribution

SMG

##### Notes

Occasional Migrant. New Azores Record

#### Falco naumanni

Fleischer, 1818

##### Ecological interactions

###### Native status

Palearctic

##### Distribution

SMG; SMR*

##### Notes

Occasional Migrant. Rodrigues et al. (2010)

#### Falco peregrinus

Tunstall, 1771

##### Ecological interactions

###### Native status

Holarctic

##### Distribution

COR; FLO; FAI; PIC*; GRA; TER; SMG; SMR

##### Notes

Occasional Migrant; Occasional Wintering. Rodrigues et al. (2010)

#### Falco rusticolus

Linnaeus, 1758

##### Ecological interactions

###### Native status

Holarctic

##### Distribution

AZO

##### Notes

Occasional Migrant. New Azores Record

#### Falco sparverius

Linnaeus, 1758

##### Ecological interactions

###### Native status

Nearctic

##### Distribution

TER; SMG

##### Notes

Occasional Migrant. Rodrigues et al. (2010)

#### Falco subbuteo

Linnaeus, 1758

##### Ecological interactions

###### Native status

Palearctic

##### Distribution

COR; SMG*

##### Notes

Occasional Migrant. Rodrigues et al. (2010)

#### Falco tinnunculus

Linnaeus, 1758

##### Ecological interactions

###### Native status

Palearctic

##### Distribution

COR; FLO; FAI; PIC; GRA; TER; SMG; SMR

##### Notes

Occasional Migrant; Occasional Wintering. Rodrigues et al. (2010)

#### Falco vespertinus

Linnaeus, 1766

##### Ecological interactions

###### Native status

Palearctic

##### Distribution

COR*; FLO; FAI; PIC; TER*; SMG; SMR*

##### Notes

Occasional Migrant. Rodrigues et al. (2010)

#### Psittaciformes

#### Psittacidae

#### Psittacula krameri

(Scopoli, 1769)

##### Ecological interactions

###### Native status

Afro-tropical

##### Distribution

FAI*; TER*; SMG (Breeder)

##### Notes

Introduced. Rodrigues et al. (2010)

#### Passeriformes

#### Laniidae

#### Lanius collurio

Linnaeus, 1758

##### Ecological interactions

###### Native status

Palearctic

##### Distribution

COR

##### Notes

Occasional Migrant. Rodrigues et al. (2010)

#### Lanius senator

Linnaeus, 1758

##### Ecological interactions

###### Native status

Palearctic

##### Distribution

SMG; SMR

##### Notes

Occasional Migrant. New Azores Record

#### Lanius excubitor borealis

Vieillot, 1808

##### Ecological interactions

###### Native status

Nearctic

##### Distribution

COR

##### Notes

Occasional Migrant. New Azores Record

#### Vireoidae

#### Vireo flavifrons

Vieillot, 1808

##### Ecological interactions

###### Native status

Nearctic

##### Distribution

COR; SMG

##### Notes

Occasional Migrant. Rodrigues et al. (2010)

#### Vireo griseus

(Boddaert, 1783)

##### Ecological interactions

###### Native status

Nearctic

##### Distribution

COR; FLO*

##### Notes

Occasional Migrant. Rodrigues et al. (2010)

#### Vireo olivaceus

(Linnaeus, 1766)

##### Ecological interactions

###### Native status

Nearctic

##### Distribution

COR; FLO; FAI*; TER*

##### Notes

Occasional Migrant. Rodrigues et al. (2010)

#### Vireo philadelphicus

(Cassin, 1851)

##### Ecological interactions

###### Native status

Nearctic

##### Distribution

COR; FLO*

##### Notes

Occasional Migrant. Rodrigues et al. (2010)

#### Oriolidae

#### Oriolus oriolus

(Linnaeus, 1758)

##### Ecological interactions

###### Native status

Palearctic

##### Distribution

COR*; FLO; SMG

##### Notes

Occasional Migrant. Rodrigues et al. (2010)

#### Corvidae

#### Coloeus monedula

(Linnaeus, 1758)

##### Ecological interactions

###### Native status

Palearctic

##### Distribution

SMG

##### Notes

Occasional Migrant. Rodrigues et al. (2010)

#### Corvus corone corone

Linnaeus, 1758

##### Ecological interactions

###### Native status

Palearctic

##### Distribution

PIC; TER; SMG

##### Notes

Occasional Migrant. Rodrigues et al. (2010)

#### Corvus frugilegus

Linnaeus, 1758

##### Ecological interactions

###### Native status

Palearctic

##### Distribution

SMG; SMR

##### Notes

Occasional Migrant. Rodrigues et al. (2010)

#### Bombycillidae

#### Bombycilla cedrorum

Vieillot, 1808

##### Ecological interactions

###### Native status

Nearctic

##### Distribution

COR; FLO

##### Notes

Occasional Migrant. New Azores Record

#### Bombycilla garrulus

(Linnaeus, 1758)

##### Ecological interactions

###### Native status

Holarctic

##### Distribution

FAI

##### Notes

Occasional Migrant. Rodrigues et al. (2010)

#### Alaudidae

#### Alauda arvensis

Linnaeus, 1758

##### Ecological interactions

###### Native status

Palearctic

##### Distribution

COR; FLO; FAI; TER*; SMG; SMR

##### Notes

Occasional Migrant. Rodrigues et al. (2010)

#### Calandrella brachydactyla

(Leisler, 1814)

##### Ecological interactions

###### Native status

Palearctic

##### Distribution

SJG; SMR*

##### Notes

Occasional Migrant. Rodrigues et al. (2010)

#### Galerida cristata

(Linnaeus, 1758)

##### Ecological interactions

###### Native status

Palearctic

##### Distribution

SMG

##### Notes

Occasional Migrant. Rodrigues et al. (2010)

#### Hirundinidae

#### Cecropis daurica

(Laxmann, 1769)

##### Ecological interactions

###### Native status

Palearctic

##### Distribution

COR*; FAI; SJG; SMG*

##### Notes

Occasional Migrant. Rodrigues et al. (2010)

#### Delichon urbicum

(Linnaeus, 1758)

##### Ecological interactions

###### Native status

Palearctic

##### Distribution

COR; FLO; FAI; PIC; GRA; SJG*; TER; SMG; SMR

##### Notes

Occasional Migrant. Rodrigues et al. (2010)

#### Hirundo rustica erythrogaster

Boddaert, 1783

##### Ecological interactions

###### Native status

Nearctic

##### Distribution

COR; FLO; TER

##### Notes

Occasional Migrant. Rodrigues et al. (2010)

#### Hirundo rustica rustica

Linnaeus, 1758

##### Ecological interactions

###### Native status

Palearctic

##### Distribution

COR; FLO; FAI; PIC*; GRA; TER; SMG; SMR

##### Notes

Occasional Migrant. Rodrigues et al. (2010)

#### Petrochelidon pyrrhonota

(Vieillot, 1817)

##### Ecological interactions

###### Native status

Nearctic

##### Distribution

COR; TER

##### Notes

Occasional Migrant. Rodrigues et al. (2010)

#### Progne subis

(Linnaeus, 1758)

##### Ecological interactions

###### Native status

Nearctic

##### Distribution

COR; FLO

##### Notes

Occasional Migrant. Rodrigues et al. (2010)

#### Riparia paludicola

(Vieillot, 1817)

##### Ecological interactions

###### Native status

Palearctic

##### Distribution

SMR

##### Notes

Occasional Migrant. New Azores Record

#### Riparia riparia

(Linnaeus, 1758)

##### Ecological interactions

###### Native status

Holarctic

##### Distribution

COR*; FLO; PIC; TER; SMG; SMR

##### Notes

Occasional Migrant. Rodrigues et al. (2010)

#### Tachycineta bicolor

(Vieillot, 1808)

##### Ecological interactions

###### Native status

Nearctic

##### Distribution

COR; FLO; SMG

##### Notes

Occasional Migrant. Rodrigues et al. (2010)

#### Phylloscopidae

#### Phylloscopus collybita

(Vieillot, 1817)

##### Ecological interactions

###### Native status

Palearctic

##### Distribution

COR; FLO; FAI; PIC; GRA*; TER; SMG; SMR

##### Notes

Occasional Migrant. Rodrigues et al. (2010)

#### Phylloscopus inornatus

(Blyth, 1842)

##### Ecological interactions

###### Native status

Palearctic

##### Distribution

PIC*; SMG

##### Notes

Occasional Migrant. Rodrigues et al. (2010)

#### Phylloscopus trochilus

(Linnaeus, 1758)

##### Ecological interactions

###### Native status

Palearctic

##### Distribution

COR; FLO; SJG; TER*; SMG

##### Notes

Occasional Migrant. Rodrigues et al. (2010)

#### Acrocephalidae

#### Acrocephalus schoenobaenus

(Linnaeus, 1758)

##### Ecological interactions

###### Native status

Palearctic

##### Distribution

COR; TER

##### Notes

Occasional Migrant. New Azores Record

#### Acrocephalus agricola

(Jerdon, 1845)

##### Ecological interactions

###### Native status

Palearctic

##### Distribution

COR

##### Notes

Occasional Migrant. Rodrigues et al. (2010)

#### Locustellidae

#### Locustella naevia

(Boddaert, 1783)

##### Ecological interactions

###### Native status

Palearctic

##### Distribution

FLO

##### Notes

Occasional Migrant. New Azores Record

#### Sylviidae

#### Hippolais polyglotta

(Vieillot, 1817)

##### Ecological interactions

###### Native status

Palearctic

##### Distribution

COR

##### Notes

Occasional Migrant. New Azores Record

#### Regulus regulus azoricus

Seebohm, 1883

##### Ecological interactions

###### Native status

Palearctic

###### Conservation status

P; A; B-II

##### Distribution

SMG (Breeder)

##### Notes

Azores Endemic. Rodrigues et al. (2010)

#### Regulus regulus inermis

Murphy & Chapin, 1929

##### Ecological interactions

###### Native status

Palearctic

###### Conservation status

P; A; B-II

##### Distribution

FLO; FAI; PIC; GRA; SJG; TER

##### Notes

Azores Endemic. Rodrigues et al. (2010)

#### Regulus regulus sanctaemariae

Vaurie, 1954

##### Ecological interactions

###### Native status

Palearctic

###### Conservation status

P; A; B-II

##### Distribution

SMR (Breeder)

##### Notes

Azores Endemic. Rodrigues et al. (2010)

#### Sylvia atricapilla gularis

Alexander, 1898

##### Ecological interactions

###### Native status

Palearctic

###### Conservation status

A; B-II

##### Distribution

COR (Breeder); FLO (Breeder); FAI (Breeder); PIC (Breeder); GRA (Breeder); SJG (Breeder); TER (Breeder); SMG (Breeder); SMR (Breeder)

##### Notes

Azores Endemic. Rodrigues et al. (2010)

#### Sylvia borin

(Boddaert, 1783)

##### Ecological interactions

###### Native status

Palearctic

##### Distribution

COR; FLO*

##### Notes

Occasional Migrant. Rodrigues et al. (2010)

#### Sylvia cantillans

(Pallas, 1764)

##### Ecological interactions

###### Native status

Palearctic

##### Distribution

COR

##### Notes

Occasional Migrant. New Azores Record

#### Sylvia nisoria

(Bechstein, 1792)

##### Ecological interactions

###### Native status

Palearctic

##### Distribution

FLO

##### Notes

Occasional Migrant. New Azores Record

#### Sylvia communis

Latham, 1787

##### Ecological interactions

###### Native status

Palearctic

##### Distribution

TER

##### Notes

Occasional Migrant. New Azores Record

#### Troglodytidae

#### Troglodytes troglodytes

(Linnaeus, 1758)

##### Ecological interactions

###### Native status

Palearctic

##### Distribution

SMG

##### Notes

Occasional Migrant. Rodrigues et al. (2010)

#### Mimidae

#### Dumetella carolinensis

(Linnaeus, 1766)

##### Ecological interactions

###### Native status

Nearctic

##### Distribution

FLO

##### Notes

Occasional Migrant. New Azores Record

#### Sturnidae

#### Sturnus vulgaris granti

Hartert, 1903

##### Ecological interactions

###### Native status

Palearctic

###### Conservation status

A-IIB

##### Distribution

COR (Breeder); FLO (Breeder); FAI (Breeder); PIC (Breeder); GRA (Breeder); SJG (Breeder); TER (Breeder); SMG (Breeder); SMR (Breeder)

##### Notes

Azores Endemic. Rodrigues et al. (2010)

#### Turdidae

#### Catharus guttatus

(Pallas, 1811)

##### Ecological interactions

###### Native status

Nearctic

##### Distribution

COR; FLO*

##### Notes

Occasional Migrant. Rodrigues et al. (2010)

#### Catharus minimus

(Lafresnaye, 1848)

##### Ecological interactions

###### Native status

Nearctic

##### Distribution

COR; FLO

##### Notes

Occasional Migrant. Rodrigues et al. (2010)

#### Catharus ustulatus

(Nuttall, 1840)

##### Ecological interactions

###### Native status

Nearctic

##### Distribution

COR; FLO

##### Notes

Occasional Migrant. New Azores Record

#### Hylocichla mustelina

(Gmelin, 1789)

##### Ecological interactions

###### Native status

Nearctic

##### Distribution

COR*; SMG

##### Notes

Occasional Migrant. Rodrigues et al. (2010)

#### Turdus iliacus

Linnaeus, 1758

##### Ecological interactions

###### Native status

Palearctic

##### Distribution

SMG; SMR

##### Notes

Occasional Migrant. Rodrigues et al. (2010)

#### Turdus merula azorensis

Hartert, 1905

##### Ecological interactions

###### Native status

Palearctic

###### Conservation status

A-IIB

##### Distribution

COR (Breeder); FLO (Breeder); FAI (Breeder); PIC (Breeder); GRA (Breeder); SJG (Breeder); TER (Breeder); SMG (Breeder); SMR (Breeder)

##### Notes

Azores Endemic. Rodrigues et al. (2010)

#### Turdus migratorius

Linnaeus, 1766

##### Ecological interactions

###### Native status

Nearctic

##### Distribution

COR

##### Notes

Occasional Migrant. New Azores Record

#### Turdus naumanni

Temminck, 1820

##### Ecological interactions

###### Native status

Palearctic

##### Distribution

SMG

##### Notes

Occasional Migrant. Rodrigues et al. (2010)

#### Turdus philomelos

Brehm, 1831

##### Ecological interactions

###### Native status

Palearctic

##### Distribution

SMG; SMR

##### Notes

Occasional Migrant. Rodrigues et al. (2010)

#### Turdus pilaris

Linnaeus, 1758

##### Ecological interactions

###### Native status

Palearctic

##### Distribution

COR; FLO; PIC*; GRA; TER; SMG; SMR

##### Notes

Occasional Migrant; Occasional Wintering. Rodrigues et al. (2010)

#### Turdus torquatus

Linnaeus, 1758

##### Ecological interactions

###### Native status

Palearctic

##### Distribution

COR; SMG*; SMR*

##### Notes

Occasional Migrant. Rodrigues et al. (2010)

#### Turdus viscivorus

Linnaeus, 1758

##### Ecological interactions

###### Native status

Palearctic

##### Distribution

SMG

##### Notes

Occasional Migrant. Rodrigues et al. (2010)

#### Muscicapidae

#### Erithacus rubecula rubecula

(Linnaeus, 1758)

##### Ecological interactions

###### Native status

Palearctic

###### Conservation status

A; B-II

##### Distribution

FAI (Breeder); PIC (Breeder); GRA (Breeder); SJG (Breeder); TER (Breeder); SMG (Breeder); SMR (Breeder)

##### Notes

Native. Rodrigues et al. (2010)

#### Ficedula hypoleuca

(Pallas, 1764)

##### Ecological interactions

###### Native status

Palearctic

##### Distribution

SMG

##### Notes

Occasional Migrant. Rodrigues et al. (2010)

#### Ficedula parva

(Bechstein, 1792)

##### Ecological interactions

###### Native status

Palearctic

##### Distribution

TER

##### Notes

Occasional Migrant. Rodrigues et al. (2010)

#### Muscicapa striata

(Pallas, 1764)

##### Ecological interactions

###### Native status

Palearctic

##### Distribution

COR; FLO; SMR

##### Notes

Occasional Migrant. New Azores Record

#### Oenanthe oenanthe leucorhoa

(Gmelin, 1789)

##### Ecological interactions

###### Native status

Nearctic

##### Distribution

COR; FLO; FAI; PIC; GRA; SJG; TER; SMG; SMR

##### Notes

Occasional Migrant. Rodrigues et al. (2010)

#### Oenanthe hispanica

(Linnaeus, 1758)

##### Ecological interactions

###### Native status

Palearctic

###### Conservation status

A; B-II

##### Distribution

FLO; SMR

##### Notes

Occasional Migrant. Rodrigues et al. (2010)

#### Oenanthe isabellina

(Temminck, 1829)

##### Ecological interactions

###### Native status

Palearctic

##### Distribution

FLO

##### Notes

Occasional Migrant. Rodrigues et al. (2010)

#### Phoenicurus ochruros

(Gmelin, 1774)

##### Ecological interactions

###### Native status

Palearctic

##### Distribution

TER*; SMG; SMR

##### Notes

Occasional Migrant. Rodrigues et al. (2010)

#### Phoenicurus phoenicurus

(Linnaeus, 1758)

##### Ecological interactions

###### Native status

Palearctic

##### Distribution

COR* (Occasional Breeder); FLO; PIC; SMG

##### Notes

Occasional Migrant; Occasional Wintering. Rodrigues et al. (2010)

#### Saxicola rubetra

(Linnaeus, 1758)

##### Ecological interactions

###### Native status

Palearctic

##### Distribution

COR*; FLO; SJG; SMG

##### Notes

Occasional Migrant. Rodrigues et al. (2010)

#### Saxicola rubicola

(Linnaeus, 1766)

##### Ecological interactions

###### Native status

Palearctic

##### Distribution

SMG

##### Notes

Occasional Migrant. Rodrigues et al. (2010)

#### Passeridae

#### Passer domesticus domesticus

(Linnaeus, 1758)

##### Ecological interactions

###### Native status

Palearctic

###### Conservation status

A

##### Distribution

COR (Breeder); FLO (Breeder); FAI (Breeder); PIC (Breeder); GRA (Breeder); SJG (Breeder); TER (Breeder); SMG (Breeder); SMR (Breeder)

##### Notes

Introduced. Rodrigues et al. (2010)

#### Petronia petronia

(Linnaeus, 1766)

##### Ecological interactions

###### Native status

Palearctic

##### Distribution

TER; SMG

##### Notes

Occasional Migrant. Rodrigues et al. (2010)

#### Estrildidae

#### Estrilda astrild

(Linnaeus, 1758)

##### Ecological interactions

###### Native status

Afro-tropical

##### Distribution

TER (Breeder); SMG (Breeder)

##### Notes

Introduced. Rodrigues et al. (2010)

#### Motacillidae

#### Anthus campestris

(Linnaeus, 1758)

##### Ecological interactions

###### Native status

Palearctic

##### Distribution

SMG

##### Notes

Occasional Migrant. Rodrigues et al. (2010)

#### Anthus cervinus

(Pallas, 1811)

##### Ecological interactions

###### Native status

Palearctic

##### Distribution

COR; TER*; SMG; SMR*

##### Notes

Occasional Migrant. Rodrigues et al. (2010)

#### Anthus pratensis

(Linnaeus, 1758)

##### Ecological interactions

###### Native status

Palearctic

##### Distribution

FLO; TER

##### Notes

Occasional Migrant. Rodrigues et al. (2010)

#### Anthus rubescens rubescens

(Tunstall, 1771)

##### Ecological interactions

###### Native status

Nearctic

##### Distribution

COR; FLO*; PIC; TER; SMG; SMR

##### Notes

Occasional Wintering. Rodrigues et al. (2010)

#### Anthus trivialis

(Linnaeus, 1758)

##### Ecological interactions

###### Native status

Palearctic

##### Distribution

COR; TER

##### Notes

Occasional Migrant. New Azores Record

#### Motacilla alba alba

Linnaeus, 1758

##### Ecological interactions

###### Native status

Palearctic

##### Distribution

COR*; FLO; GRA; TER; SMG; SMR

##### Notes

Occasional Migrant. Rodrigues et al. (2010)

#### Motacilla cinerea patriciae

Vaurie, 1957

##### Ecological interactions

###### Native status

Palearctic

###### Conservation status

A; B-II

##### Distribution

COR (Breeder); FLO (Breeder); FAI (Breeder); PIC (Breeder); GRA (Breeder); SJG (Breeder); TER (Breeder); SMG (Breeder); SMR (Breeder)

##### Notes

Azores Endemic. Rodrigues et al. (2010)

#### Motacilla citreola

Pallas, 1776

##### Ecological interactions

###### Native status

Palearctic

##### Distribution

COR; SMR*

##### Notes

Occasional Migrant. Rodrigues et al. (2010)

#### Motacilla flava

Linnaeus, 1758

##### Ecological interactions

###### Native status

Palearctic

##### Distribution

COR; PIC; TER*; SMG; SMR

##### Notes

Occasional Migrant. Rodrigues et al. (2010)

#### Fringillidae

#### Acanthis flammea

(Linnaeus, 1758)

##### Ecological interactions

###### Native status

Holarctic

##### Distribution

COR; FLO; SMG

##### Notes

Occasional Migrant. Rodrigues et al. (2010)

#### Acanthis hornemanni

(Holböll, 1843)

##### Ecological interactions

###### Native status

Holarctic

##### Distribution

COR

##### Notes

Occasional Migrant. Rodrigues et al. (2010)

#### Carduelis carduelis parva

Tschusi, 1901

##### Ecological interactions

###### Native status

Palearctic

###### Conservation status

A; B-II

##### Distribution

COR (Breeder); FLO (Breeder); FAI (Breeder); PIC (Breeder); GRA (Breeder); SJG (Breeder); TER (Breeder); SMG (Breeder); SMR (Breeder)

##### Notes

Macaronesian Endemic. Rodrigues et al. (2010)

#### Chloris chloris aurantiiventris

(Cabanis, 1851)

##### Ecological interactions

###### Native status

Palearctic

###### Conservation status

A; B-II

##### Distribution

FAI*; PIC*; TER (Breeder); SMG (Breeder); SMR*

##### Notes

Introduced. Rodrigues et al. (2010)

#### Fringilla coelebs moreletti

Pucheran, 1859

##### Ecological interactions

###### Native status

Palearctic

###### Conservation status

A

##### Distribution

COR (Breeder); FLO (Breeder); FAI (Breeder); PIC (Breeder); GRA (Breeder); SJG (Breeder); TER (Breeder); SMG (Breeder); SMR (Breeder)

##### Notes

Azores Endemic. Rodrigues et al. (2010)

#### Fringilla montifringilla

Linnaeus, 1758

##### Ecological interactions

###### Native status

Palearctic

##### Distribution

SMR

##### Notes

Occasional Wintering. New Azores Record

#### Linaria cannabina

(Linnaeus, 1758)

##### Ecological interactions

###### Native status

Palearctic

##### Distribution

TER*; SMG

##### Notes

Occasional Migrant. Rodrigues et al. (2010)

#### Linaria flavirostris

(Linnaeus, 1758)

##### Ecological interactions

###### Native status

Palearctic

##### Distribution

FLO

##### Notes

Occasional Migrant. New Azores Record

#### Loxia curvirostra

Linnaeus, 1758

##### Ecological interactions

###### Native status

Holarctic

##### Distribution

SMG

##### Notes

Occasional Migrant. Rodrigues et al. (2010)

#### Pyrrhula murina

Godman, 1866

##### Ecological interactions

###### Native status

Palearctic

###### Conservation status

P; A-I; T100

##### Distribution

SMG (Breeder)

##### Notes

Azores Endemic. Rodrigues et al. (2010)

#### Serinus canaria

(Linnaeus, 1758)

##### Ecological interactions

###### Native status

Palearctic

###### Conservation status

A

##### Distribution

COR (Breeder); FLO (Breeder); FAI (Breeder); PIC (Breeder); GRA (Breeder); SJG (Breeder); TER (Breeder); SMG (Breeder); SMR (Breeder)

##### Notes

Macaronesian Endemic. Rodrigues et al. (2010)

#### Serinus serinus

(Linnaeus, 1766)

##### Ecological interactions

###### Native status

Palearctic

##### Distribution

TER

##### Notes

Occasional Migrant. Rodrigues et al. (2010)

#### Spinus spinus

(Linnaeus, 1758)

##### Ecological interactions

###### Native status

Palearctic

##### Distribution

COR; FLO; SMG

##### Notes

Occasional Migrant; Occasional Wintering. Rodrigues et al. (2010)

#### Parulidae

#### Cardellina canadensis

(Linnaeus, 1766)

##### Ecological interactions

###### Native status

Nearctic

##### Distribution

COR

##### Notes

Occasional Migrant. Rodrigues et al. (2010)

#### Geothlypis trichas

(Linnaeus, 1766)

##### Ecological interactions

###### Native status

Nearctic

##### Distribution

COR; FLO

##### Notes

Occasional Migrant. Rodrigues et al. (2010)

#### Leiothlypis peregrina

(Wilson, 1811)

##### Ecological interactions

###### Native status

Nearctic

##### Distribution

COR; FLO*

##### Notes

Occasional Migrant. Rodrigues et al. (2010)

#### Mniotilta varia

(Linnaeus, 1766)

##### Ecological interactions

###### Native status

Nearctic

##### Distribution

COR; FLO*

##### Notes

Occasional Migrant. Rodrigues et al. (2010)

#### Parkesia noveboracensis

(Gmelin, 1789)

##### Ecological interactions

###### Native status

Nearctic

##### Distribution

COR*; SMG*; SMR

##### Notes

Occasional Migrant. Rodrigues et al. (2010)

#### Setophaga americana

(Linnaeus, 1758)

##### Ecological interactions

###### Native status

Nearctic

##### Distribution

COR; FLO*

##### Notes

Occasional Migrant. Rodrigues et al. (2010)

#### Seiurus aurocapilla

(Linnaeus, 1766)

##### Ecological interactions

###### Native status

Nearctic

##### Distribution

COR; FLO*; TER

##### Notes

Occasional Migrant. Rodrigues et al. (2010)

#### Setophaga caerulescens

(Gmelin, 1789)

##### Ecological interactions

###### Native status

Nearctic

##### Distribution

COR

##### Notes

Occasional Migrant. Rodrigues et al. (2010)

#### Setophaga citrina

(Boddaert, 1783)

##### Ecological interactions

###### Native status

Nearctic

##### Distribution

COR

##### Notes

Occasional Migrant. Rodrigues et al. (2010)

#### Setophaga coronata

(Linnaeus, 1766)

##### Ecological interactions

###### Native status

Nearctic

##### Distribution

COR; FLO; SMG

##### Notes

Occasional Migrant. Rodrigues et al. (2010)

#### Setophaga discolor

(Vieillot, 1809)

##### Ecological interactions

###### Native status

Nearctic

##### Distribution

COR

##### Notes

Occasional Migrant. New Azores Record

#### Setophaga dominica

(Linnaeus, 1766)

##### Ecological interactions

###### Native status

Nearctic

##### Distribution

COR

##### Notes

Occasional Migrant. New Azores Record

#### Setophaga magnolia

(Wilson, 1811)

##### Ecological interactions

###### Native status

Nearctic

##### Distribution

COR*; FLO

##### Notes

Occasional Migrant. Rodrigues et al. (2010)

#### Setophaga pensylvanica

(Linnaeus, 1766)

##### Ecological interactions

###### Native status

Nearctic

##### Distribution

COR

##### Notes

Occasional Migrant. Rodrigues et al. (2010)

#### Setophaga petechia

(Linnaeus, 1766)

##### Ecological interactions

###### Native status

Nearctic

##### Distribution

COR; FLO; SMG; SMR*

##### Notes

Occasional Migrant. Rodrigues et al. (2010)

#### Setophaga ruticilla

(Linnaeus, 1758)

##### Ecological interactions

###### Native status

Nearctic

##### Distribution

COR; FLO*

##### Notes

Occasional Migrant. Rodrigues et al. (2010)

#### Setophaga striata

(Forster, 1772)

##### Ecological interactions

###### Native status

Nearctic

##### Distribution

COR; FLO; SMG

##### Notes

Occasional Migrant. Rodrigues et al. (2010)

#### Setophaga virens

(Gmelin, 1789)

##### Ecological interactions

###### Native status

Nearctic

##### Distribution

COR

##### Notes

Occasional Migrant. Rodrigues et al. (2010)

#### Vermivora chrysoptera

(Linnaeus, 1766)

##### Ecological interactions

###### Native status

Nearctic

##### Distribution

COR

##### Notes

Occasional Migrant. New Azores Record

#### Vermivora cyanoptera

Olson & Reveal, 2009

##### Ecological interactions

###### Native status

Nearctic

##### Distribution

COR

##### Notes

Occasional Migrant. New Azores Record

#### Icteridae

#### Dolichonyx oryzivorus

(Linnaeus, 1758)

##### Ecological interactions

###### Native status

Nearctic

##### Distribution

COR; FLO; SMR*

##### Notes

Occasional Migrant. Rodrigues et al. (2010)

#### Icterus galbula

(Linnaeus, 1758)

##### Ecological interactions

###### Native status

Nearctic

##### Distribution

COR; FLO; SMR*

##### Notes

Occasional Migrant. Rodrigues et al. (2010)

#### Emberizidae

#### Emberiza rustica

Pallas, 1776

##### Ecological interactions

###### Native status

Palearctic

##### Distribution

SMR

##### Notes

Occasional Migrant. New Azores Record

#### Junco hyemalis

(Linnaeus, 1758)

##### Ecological interactions

###### Native status

Nearctic

##### Distribution

FLO

##### Notes

Occasional Migrant. Rodrigues et al. (2010)

#### Melospiza lincolnii

(Audubon, 1834)

##### Ecological interactions

###### Native status

Nearctic

##### Distribution

COR

##### Notes

Occasional Migrant. New Azores Record

#### Passerculus sandwichensis

(Gmelin, 1789)

##### Ecological interactions

###### Native status

Nearctic

##### Distribution

COR; FLO

##### Notes

Occasional Migrant. Rodrigues et al. (2010)

#### Passerina cyanea

(Linnaeus, 1766)

##### Ecological interactions

###### Native status

Nearctic

##### Distribution

COR; FLO; SMR*

##### Notes

Occasional Migrant. Rodrigues et al. (2010)

#### Zonotrichia albicollis

(Gmelin, 1789)

##### Ecological interactions

###### Native status

Nearctic

##### Distribution

COR; SMR*

##### Notes

Occasional Migrant. New Azores Record

#### Zonotrichia leucophrys

(Forster, 1772)

##### Ecological interactions

###### Native status

Nearctic

##### Distribution

COR; FLO; SMR*

##### Notes

Occasional Migrant. Rodrigues et al. (2010)

#### Calcariidae

#### Calcarius lapponicus

(Linnaeus, 1758)

##### Ecological interactions

###### Native status

Holarctic

##### Distribution

COR; FLO; TER; SMG

##### Notes

Occasional Migrant. Rodrigues et al. (2010)

#### Plectrophenax nivalis

(Linnaeus, 1758)

##### Ecological interactions

###### Native status

Holarctic

##### Distribution

COR; FLO; FAI; PIC; GRA; TER; SMG; SMR

##### Notes

Occasional Migrant; Occasional Wintering. Rodrigues et al. (2010)

#### Cardinalidae

#### Pheucticus ludovicianus

(Linnaeus, 1766)

##### Ecological interactions

###### Native status

Nearctic

##### Distribution

COR; FLO; SMG; SMR*

##### Notes

Occasional Migrant. Rodrigues et al. (2010)

#### Piranga olivacea

(Gmelin, 1789)

##### Ecological interactions

###### Native status

Nearctic

##### Distribution

COR; FLO

##### Notes

Occasional Migrant. Rodrigues et al. (2010)

#### Piranga rubra

(Linnaeus, 1758)

##### Ecological interactions

###### Native status

Nearctic

##### Distribution

COR

##### Notes

Occasional Migrant. Rodrigues et al. (2010)

#### Spiza americana

(Gmelin, 1789)

##### Ecological interactions

###### Native status

Nearctic

##### Distribution

COR*; FLO; SMR*

##### Notes

Occasional Migrant. Rodrigues et al. (2010)

### List of potential breeding species from Azores

#### Chordata

#### Vertebrata

#### Aves

#### Anseriformes

#### Anatidae

#### Aix galericulata

(Linnaeus, 1758)

##### Ecological interactions

###### Native status

Palearctic

##### Distribution

PIC*; GRA; TER; SMG

##### Notes

Occasional Migrant. Rodrigues et al. (2010)

#### Galliformes

#### Numididae

#### Numida meleagris

(Linnaeus, 1758)

##### Ecological interactions

###### Native status

Afro-tropical

##### Distribution

SMG

##### Notes

Introduced. Rodrigues et al. (2010)

#### Odontophoridae

#### Colinus virginianus

(Linnaeus, 1758)

##### Ecological interactions

###### Native status

Nearctic

##### Distribution

FAI

##### Notes

Introduced. Rodrigues et al. (2010)

#### Phasianidae

#### Perdix perdix

(Linnaeus, 1758)

##### Ecological interactions

###### Native status

Palearctic

###### Conservation status

A-IIA

##### Distribution

SMG

##### Notes

Introduced. Rodrigues et al. (2010)

#### Phasianus colchicus

Linnaeus, 1758

##### Ecological interactions

###### Native status

Palearctic

##### Distribution

AZO

##### Notes

Introduced. Rodrigues et al. (2010)

#### Passeriformes

#### Corvidae

#### Cyanocitta cristata

(Linnaeus, 1758)

##### Ecological interactions

###### Native status

Nearctic

##### Distribution

SMG

##### Notes

Introduced. Rodrigues et al. (2010)

#### Estrildidae

#### Estrilda troglodytes

(Lichtenstein, 1823)

##### Ecological interactions

###### Native status

Afro-tropical

##### Distribution

SMG

##### Notes

Introduced. Rodrigues et al. (2010)

#### Fringillidae

#### Crithagra mozambica

(Müller, 1776)

##### Ecological interactions

###### Native status

Afro-tropical

##### Distribution

TER

##### Notes

Introduced. Rodrigues et al. (2010)

## Analysis

The updated list of the bird species recorded in the Azores comprised 38 newly recorded species, increasing the total number of recorded species and subspecies to 414. These 414 taxa belong to 24 orders, 67 families, 202 genera and 404 species. Some of the species and genus names from the previous list were changed and some families were moved to other orders. The taxonomic revision results in the addition of four new orders to the checklist.

São Miguel Island stands out as the richest island (299 spp. and sspp.), followed by Terceira (248 spp. and sspp.), Corvo and Flores (238 and 232 spp. and sspp., respectively) (Fig. 4). The islands of Corvo (western group) and Santa Maria (eastern group) were the ones with the highest increases in species number (plus 14,1% and 12,%, respectively).

**Figure 5.**
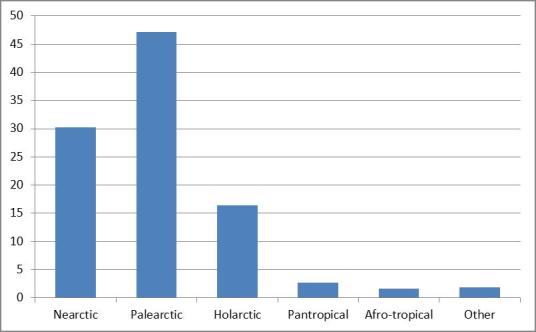
Biogeographical origin of the species present in Azores archipelago; “Other” – marine regions (Indo-Pacific; Southern Atlantic; Sub-Antarctic; Antarctic; Tropical Atlantic) (Suppl. material 2).

Almost half of the species and subspecies that occur in the Azores come from the Palearctic (n = 195, that is, 47% of spp and sspp) but the Nearctic is also well represented (n = 125 i.e., 30% of spp and sspp). The species and subspecies of Holarctic origin totalled 68 (i.e., 17% of spp and sspp) (Fig. 5). The Passeriformes (songbirds), Charadriiformes (waders, skuas, gulls, terns and auks) and Anseriformes (swans, geese and ducks) are by far the most represented orders with 129, 111 and 42 taxa, respectively.

## Discussion

The most striking feature of this checklist is that it shows not only an impressive increase in the number of new species records for the Azores (36, that is, plus 8,7% in less than five years), but also an enlargement of the distribution of several species among the different Azorean islands. A simple explanation for this could be the increase in the number of observers performing birdwatching out of the “high season” (autumn and spring) for vagrant birds, and possibly also the creation of the “Aves dos Açores” website, whose aim is to provide information about all common species and also about rarities, even though is mostly aimed at a non-birdwatcher public.

The situation of the Azores in the middle of the North Atlantic makes these islands a safe place for the birds taken away from their normal migratory routes by strong winds or by changes in wind direction (vagrants) (Liechti 2006). Vagrant birds have been observed using trans-oceanic ships to rest until they detect a landmass at close range and usually fly towards it (Lees and Gilroy 2009). The higher number of Nearctic species reported on Corvo Island could be related to the relative proximity of this island to the American continent, allowing birds to wander into it. Another explanation for this may be the combination of a very small area (17 km2) and the high observation pressure during the autumn, when several tens of birdwatchers visit the island. On the opposite side of the archipelago, Santa Maria Island, the closest island to the European continent, holds a higher proportion of Palearctic species. The comparatively low number of non-breeding species observed on Graciosa and São Jorge islands may be explained, at least partly, by a lower observation pressure than on the other islands.

The native birds from the Azores provide good examples of insular speciation, since two endemic species and 11 endemic subspecies are known. Moreover, many more endemic terrestrial species have gone extinct (Rando et al. 2013, Alcover et al. 2015), possibly as a consequence of direct predation by man, the introduction of non-native predatory species (e.g., cats, rats, ferrets and weasels), and habitat degradation or loss. The current endemic species and subspecies are the survivors of almost 600 years of human occupation and major land-use changes. The recent population recovery of the Azores Bullfinch (Fig. 2) in the north-eastern part of São Miguel is considered as a good example of the best practices of LIFE conservation programs (Ceia et al. 2010). Another example of successful conservation measures comes from the rabbit eradication and habitat restoration on Praia islet (Graciosa Island), which resulted in a significant increase in the breeding numbers of Monteiro's Storm-petrels, Madeiran Storm-petrels and terns (Bolton et al. 2004, Bried et al. 2009, Bried and Neves in press).

Species lists such as this one are important to draw attention to particular taxonomic groups, to serve as a baseline in long-term systematic monitoring, to elucidate patterns of species diversity and distribution, to elucidate information gaps about distribution, and can serve as a tool in educational projects. The currently ongoing initiatives concerning citizen ornithological science in the Azores will be of high value for the knowledge of the biology and phenology of migratory birds and will hopefully increase the pressure towards the definition and preservation of more Important Bird Areas (IBAs) in the archipelago.

## Supplementary Material

Supplementary material 1 Global species richness on the nine Azorean islands Data type: Species distribution
Brief description: Global species richness on the nine Azorean islands (COR= Corvo; FLO = Flores; FAI = Faial; PIC = Pico; GRA =Graciosa; SJG = São Jorge; TER = Terceira; SMG = São Miguel; SMR = Santa Maria).
File: oo_62386.xlsx Barcelos et al.

Supplementary material 2 Biogeographical origin of the species present in Azores archipelago Data type: occurences
Brief description: Biogeographical origin of the species present in Azores archipelago.
“Other” refers to marine regions only represented once (Indo-Pacific; Southern Atlantic; Sub-Antarctic; Antarctic; Tropical Atlantic).
File: oo_62383.xlsx Barcelos et al.

Supplementary material 3 List of Rare Species Data type: occurences
Brief description: Species with five or less records and individuals counts, in the Azores, with some notes.
File: oo_62653.xlsx Barcelos et al.
